# Perineuronal nets support astrocytic ion and glutamate homeostasis at tripartite synapses

**DOI:** 10.21203/rs.3.rs-2501039/v1

**Published:** 2023-02-02

**Authors:** Bhanu P. Tewari, AnnaLin M. Woo, Courtney E. Prim, Lata Chaunsali, Ian F. Kimbrough, Kaliroi Engel, Jack L. Browning, Susan L. Campbell, Harald Sontheimer

**Affiliations:** 1 Department of Neuroscience, University of Virginia School of Medicine, Charlottesville, VA, USA; 2 School of Neuroscience, Virginia Tech, Blacksburg, VA, USA; 3 Department of Animal Sciences, Virginia Tech, Blacksburg VA, USA

## Abstract

Perineuronal nets (PNNs) are dense, negatively charged extracellular matrices that cover the cell body of fast-spiking inhibitory neurons. Synapses can be embedded and stabilized by PNNs believed to prevent synaptic plasticity. We find that in cortical fast-spiking interneurons synaptic terminals localize to perforations in the PNNs, 95% of which contain either excitatory or inhibitory synapses or both. The majority of terminals also colocalize with astrocytic processes expressing Kir4.1 as well as glutamate (Glu) and GABA transporters, hence can be considered tripartite synapses. In the adult brain, degradation of PNNs does not alter axonal terminals but causes expansion of astrocytic coverage of the neuronal somata. However, loss of PNNs impairs astrocytic transmitter and K+ uptake and causes spillage of synaptic Glu into the extrasynaptic space. This data suggests a hitherto unrecognized role of PNNs, to synergize with astrocytes to contain synaptically released signals.

## Introduction:

With the discovery that up to 99% of excitatory synapses are associated with astrocytic processes[1], the classical view of the synapse as being formed only by two neurons has changed dramatically. Often called the “tripartite synapse”, this arrangement allows for astrocytes to sense synaptic activity and potentially modulate activity via the release of gliotransmitters[2]. Importantly, at the tripartite synapse astrocytic processes are at the perfect place to remove neurotransmitters and ions released in conjunction with synaptic activity. Astrocytic ensheathment of glutamatergic synapses is particularly important to ensure that glutamate (Glu) does not spill out of synapses and activate extra-synaptic receptors[3] as this can cause excitotoxicity[4].

Neurons and astrocytes are also embedded in an extracellular matrix (ECM) made up of proteoglycans, glycoproteins, and polysaccharides. Neurons and astrocytes each synthesize defined ECM constituents including hyaluronan (HA), chondroitin sulfate proteoglycans (CSPGs) such as aggrecan, brevican, versican and neurocan, Tenascin R (Tn-R), and link proteins such as Crtl1 and Bral2[5, 6]. Specific ECM constituents have been shown to directly interact with synaptic receptors and ion channels [7–9] thereby affecting synaptic vesicle release[10], dendritic spine morphology [11–14], and the structural integrity of synapses[13, 15]. Moreover, due to the negative charges associated with sulfate groups on the CSPGs, the ECM has been implicated in altering diffusion of ions in the extracellular space [16]as well as binding water molecules.

On some cortical inhibitory neurons, particularly those expressing the Ca^2^+ binding protein parvalbumin (PV), the ECM forms a highly condensed corset-like structure known as perineuronal nets (PNNs). Easily recognized by the binding of wisteria floribunda agglutinin (WFA) [17], PNNs encapsulate the cell soma, dendrites, and axon initial segment. PNNs have been shown to stabilize synapses and restrict synaptic plasticity, particularly in pathways that show activity-dependent plasticity during development such as the visual system[18]. However, whether and how PNNs interact structurally and functionally with astrocytes at tripartite synapses is unknown.

In the present study, we show that synapses onto PNN-expressing fast-spiking neurons (FSNs) exist in small perforations or holes within the PNN and these contain excitatory and inhibitory synapses in conjunction with astrocytic processes, hence can be considered tripartite synapses. Upon PNNs disruption, the synapses retain their place on the neuron but the astrocytes expand their coverage to vacated areas on the cell body. Importantly, we show that PNN disruption impedes astrocytic uptake of synaptically released Glu and K^+^ and causes the spillage of glutamate into the perisynaptic space. This suggests a hitherto unrecognized function of the PNNs namely to create a barrier that limits diffusion of Glu and K^+^ from the synaptic cleft so as to synergize with astrocytes for effective reuptake of neuronally released ions and transmitters. Hence PNNs are an important structural and functional contributor to the tripartite synapse.

## Results:

### 1. Astrocyte-PNN spatial interface:

Tripartite synapses on excitatory neurons are typically on spines that are almost completely ensheathed by astrocytic processes also called leaflets[19]. The near complete coverage of the synapse by astrocytes facilitates effective clearance of synaptically released ions and neurotransmitters. On inhibitory neurons, the PNN forms a coat around the cell body, some of its dendrites, and the axon initial segment. Small holes or perforations of the PNN provide access for synaptic terminals. Hence our first question was whether these axosomatic synapses on inhibitory neurons are tripartite synapses, i.e., contain astrocytic processes associated with the synapse.

**Fig. 1:**
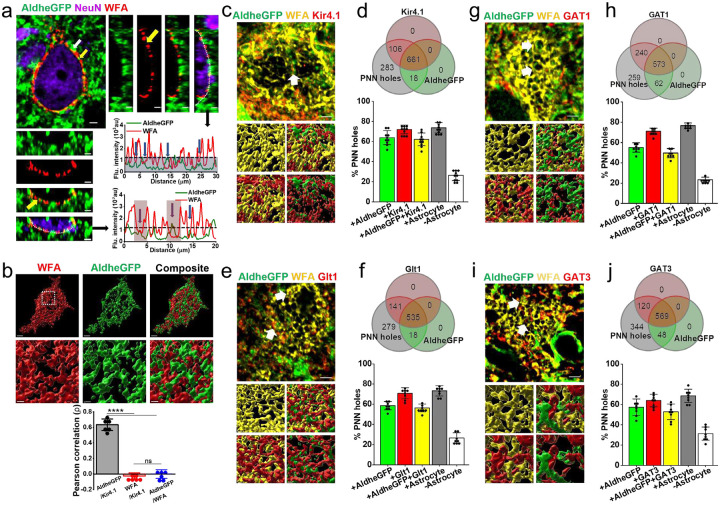
Astrocytic processes and homeostatic proteins (Kir41. And GLT-1) are largely confined to PNN holes. **a** Confocal micrograph showing WFA (red)-labelled PNN surrounded by AldheGFP (green) labelled astrocytic processes. White and yellow arrows point to astrocytic process on outer surface and within PNN holes respectively. The side and bottom panels show orthogonal planes of the 3D image. WFA and AldheGFP fluorescence intensities of a line drawn on the PNNs in orthogonal view planes showing PNN holes occupied by astrocytic processes (blue arrows). The blue area below the dotted line represents WFA threshold intensity. Vertical red bars in between two consecutive WFA peaks represent the area of PNN holes wherein astrocytic processes can be confined. Scale 2μm. **b** 3D reconstruction of PV neuron surface area occupied by PNN (WFA - red) and astrocytic processes (AldheGFP - green) showing a non-overlapping interdigitating spatial interface. The marked area in the white square is magnified in the bottom panels. Scale bar 2μm on top and 1μm on bottom panels. Error bars show a highly positive Pearson correlation between astrocytic markers AldheGFP and Kir4.1 (0.63 ± 0.07) and a significantly negative correlation between PNN marker (WFA) and astrocytic markers Kir4.1 (−0.02 ± 0.04) and AldheGFP (−0.001 ± 0.05). n = 6PNNs/3m; ****P < 0.0001; one-way ANOVA, Tukey’s post-hoc test. **c** Confocal micrograph (top) showing astrocytic processes (AldheGFP - green) expressing Kir4.1(red) in PNN (WFA - yellow) holes. Bottom panels show a 3D reconstruction of PNN holes (white arrows) occupied by astrocytic processes expressing Kir4.1. **d** Venn diagram showing proportional occupancy of PNN holes by Kir4.1-expressing astrocytic processes. Bar diagram showing the percent of total PNN holes occupied by AldheGFP (63.83 ± 7.0), Kir4.1 (71.9 ± 4.22), both (62.13 ± 6.33), occupied by any astrocytic marker (73.64 ± 5.14), and not occupied by any astrocytic marker (26.35 ± 5.14). n = >40PNNs/10s/4m. **e** Confocal micrograph (top) showing astrocytic processes (AldheGFP - green) expressing Glt1 (red) in PNN (WFA - yellow) holes. Bottom panels show 3D reconstruction of PNN holes (white arrows) occupied by astrocytic processes expressing Glt1. **f** Venn diagram showing proportional occupancy of PNN holes by Glt1 expressing astrocytic processes. Bar diagram showing the percent of total PNN holes occupied by AldheGFP (58.68 ± 4.02), Glt1 (70.85 ± 5.55), both (56.35 ± 3.87), occupied by any astrocytic marker (73.37 ± 5.11), and not occupied by any astrocytic marker (26.62 ± 5.11). n = >40PNNs/8s/4m. **g** Confocal micrograph (top) showing astrocytic processes expressing AldheGFP (green) and GAT1 (red) in PNN (WFA - yellow) holes. Bottom panels show 3D reconstruction of PNN holes occupied by astrocytic processes expressing GAT1 (red). **h** Venn diagram showing proportional occupancy of PNN holes by GAT1 expressing astrocytic processes. Bar diagram showing the percent of total PNN holes occupied by AldheGFP (55.11 ± 4.59), GAT1 (71.06 ± 3.18), both (49.62 ± 4.56), occupied by any astrocytic marker (76.64 ± 2.57), and not occupied by any astrocytic marker (23.35 ± 2.57). n = >40PNNs/8s/4m. **i** Confocal micrograph showing astrocytic processes expressing AldheGFP (green) and GAT3 (red) in PNN (WFA - yellow) holes. Bottom panels show 3D reconstruction of PNN holes occupied by astrocytic processes expressing GAT3. **j** Venn diagram showing proportional occupancy of PNN holes by GAT3 expressing astrocytic processes. Bar diagram showing the percent of total PNN holes occupied by AldheGFP (57.41 ± 8.14), GAT3 (64.0 ± 6.18), both (52.95 ± 7.74), occupied by any astrocytic marker (68.62 ± 6.48), and not occupied by any astrocytic marker (31.37 ± 6.48). n = >40PNNs/8s/4m. **s** and m represent the number of sections and mice respectively. Bar data are expressed as mean ± standard deviation (SD). Dots in the bars represent individual data points. Scale bar 2μm in top and 1μm in bottom images in c, e g, and i.

To visualize astrocytic processes in PNN holes, we used FVB-N//Swiss Webster-Aldh1l1-eGFP mice expressing eGFP in astrocytes [20] to image astrocytes in relationship to WFA-labeled PNNs in layers 3 – 4 of the somatosensory cortex. This is an area of the highest PNN density where nearly all astrocytes are contacting PNNs. Confocal images show a majority of astrocytic processes terminating within PNN holes however, some astrocytic processes terminated on the outer side of the PNN (white and yellow arrows Fig. 1a). We rarely observed astrocytic processes interspaced between the PNN and neuronal cell body. To quantify these images, we generated intensity profiles along the PNNs (Fig. 1a, dotted line). These profiles show peaks of astrocytic AldheGFP (Fig. 1a, green line) in the holes of PNNs (with lowest WFA signal, red line) suggesting astrocytes preferentially occupy PNN holes (line graphs in Fig. 1a). A 3D rendering (Fig. 1b, images) and a Pearson correlation analysis of the PNN marker WFA with astrocytic AldheGFP and Kir4.1 (Fig. 1b bar graph), shows no correlation between WFA and both astrocytic markers consistent with a non-overlapping interdigitating spatial interface where astrocytic processes are exclusively found in the PNN holes.

Less condensed PNNs are also found in small populations of excitatory neurons, for example, CA2 pyramidal neurons. To ask whether the presence of PNNs would similarly place astrocytic processes into PNN holes on excitatory cells we repeated the above analysis on sections of hippocampus CA2. Indeed, CA2 PNN holes were also occupied by astrocytic processes similar to the cortical PNNs around PV neurons (Fig. S1a, b), however with much closer spatial proximity as seen by a positive Pearson’s coefficient in both stratum pyramidale (Fig. S1c) and condensed interstitial matrix in the stratum oriens (Fig. S1d).

Since astrocytic processes are primarily confined to the PNN holes, it is reasonable to assume that these holes are sites where astrocytes perform their homeostatic and neuromodulatory functions analogous to the conventional tripartite synapses. Hence, we would expect astrocytic processes in PNN holes to express the necessary proteins associated with potassium, glutamate, and GABA uptake.

Using IHC followed by high magnification confocal imaging and line intensity profile analysis of the PNNs[21, 22], we assessed the expression of Kir4.1, Glt1, and GABA transporters GAT1 and GAT3 in combination with the astrocytic marker AldheGFP in >900 holes in each experimental group. We observed immunoreactivity of AldheGFP, Kir4.1, and both in 63%, 71%, and 62% of PNN holes respectively (Fig. 1c, d). In a separate set of experiments, we observed 59%, 70%, and 56% PNN holes with aldheGFP, Glt1, and both immunoreactivities respectively (Fig. 1e, f). In similar proportions, astrocytic processes expressed both GABA transporters, GAT1 (Fig. 1g, h) and GAT3 (Fig. 1i, j) in the PNN holes. We also examined the expression of Aqp4, Cnx43, and Cnx30, however, these all showed low to almost undetectable immunoreactivity in the PNN holes (Fig. S1e – j). These data suggest that astrocytic processes in PNN holes express the necessary proteins to support the clearance of synaptically released ions and transmitters.

### 2. PNN holes house tripartite synapses:

Since PNN holes provide access for the placement of axosomatic synapses on FSNs, we assessed whether synaptic terminals and astrocytic processes coexist in the PNN holes analogous to a classic tripartite synapse. As an extension, we wondered whether a given hole exclusively houses excitatory or inhibitory synapses and if so whether astrocytes show matching GABA or Glu transporters.

**Fig. 2:**
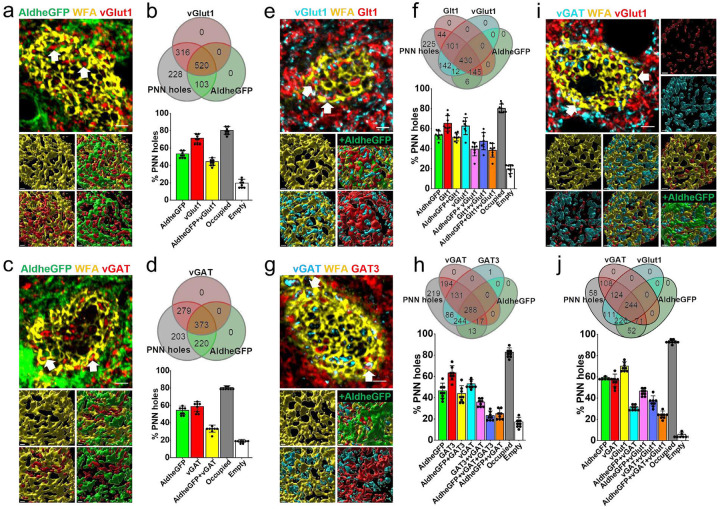
PNN holes contain excitatory and inhibitory synaptic terminals together with astrocytic processes that express corresponding neurotransmitter transporters. **a** Representative confocal micrographs showing PNN (WFA - yellow), astrocytic processes (AldheGFP - green), and excitatory presynaptic terminals (vGlut1 - red) expression around cortical FSNs. Arrows point to the PNN holes containing both vGlut1 and AldheGFP. Bottom images represent 3D reconstruction of the PNN lattice, containing astrocytic processes and vGlut1 terminals. **b** Venn diagram showing the proportional occupancy of PNN holes by vGlut1 expressing excitatory synapses and AldheGFP expressing astrocytic processes. Bar diagram showing the percent of total PNN holes occupied by AldheGFP (53.28 ± 4.05), vGlut1 (71.37 ± 3.18), and both (44.45 ± 4.26). 80.39 ± 4.46 *%* of total holes are occupied (by AldheGFP or vGlut1 or both) leaving 19.60 ± 4.4 % of holes empty. n = >40PNNs/8s/4m. **c** Representative confocal micrographs showing PNN (WFA - yellow), astrocytic processes (AldheGFP - green), and inhibitory presynaptic terminals (vGAT - red) expression around cortical FSNs. Arrows point to the PNN holes containing both vGAT and AldheGFP. Bottom images represent 3D reconstruction of the PNN lattice containing astrocytic processes and vGAT terminals. **d** Venn diagram showing the proportional occupancy of PNN holes by astrocytic processes and GABAergic synapses. Bar diagram showing the percent of total PNN holes occupied by AldheGFP (54.45 ± 4.7), vGAT (58.74 ± 5.5), and both (33.31 ± 4.02). 80.89 ± 1.8 % of total holes are occupied (by AldheGFP or vGAT or both) leaving 19.10 ± 1.8 % holes empty. n = >40PNNs/8s/4m. **e** Representative confocal micrographs showing PNN (WFA - yellow), astrocytic processes labelled with AldheGFP (green) and Glt1 (red), and excitatory presynaptic terminals (vGlut1 - cyan) expression around cortical FSNs. Arrows point to the PNN holes containing both Glt1 and vGlut1. Bottom images represent 3D reconstruction of PNN lattice containing astrocytic processes and vGAT terminals. **f** Venn diagram showing the proportional occupancy of PNN holes by glutamatergic synapses and astrocytic processes with glutamate transporter expression. Bar diagram shows the percent of total PNN holes occupied by AldheGFP (53.73 ± 4.08), Glt1 (65.36 ± 7.83), AldheGFP + Glt1 (51.81 ± 3.87), vGlut1 (62.72 ± 8.45), AldheGFP + vGlut1 (39.68 ± 6.76), Glt1 + vGlut1 (47.56 ± 8.55), and AldheGFP + Glt1 + vGlut1 (38.44 ± 6.79). 80.31 ± 4.4 % of total holes are occupied (by astrocytic markers or synapses or both) leaving 19.68 ± 4.4 % holes empty. n = >40PNNs/8s/4m. **g** Representative confocal micrographs showing PNN (WFA - yellow), astrocytic processes labelled with AldheGFP (green) and GAT3 (red), and inhibitory presynaptic terminals (vGAT - cyan) expression around cortical PV neuron. Arrows point to the PNN holes containing both GAT3 and vGAT. Bottom images represent 3D reconstruction of PNN showing PNN holes containing astrocytic processes with GABA transporter and GABAergic synapses. **h** Venn diagram showing the proportional occupancy of PNN holes by GABAergic synapses and astrocytic processes with GABA transporter expression. Bar diagram shows the percent of total PNN holes occupied by AldheGFP (46.51 ± 7.17), GAT3 (63.37 ± 6.81), AldheGFP + GAT3 (43.89 ± 7.4), vGAT (53.11 ± 3.99), GAT3 + vGAT (35.89 ± 3.63), AldheGFP + vGAT + GAT3 (23.12 ± 4.45), and AldheGFP + vGAT (25.02 ± 4.76). 82.72 ± 4.2 % of total holes are occupied (by astrocytic markers or synapses or both) leaving 17.27 ± 4.2 % holes empty. n = >40PNNs/8s/4m. **i** Representative confocal micrographs showing PNN (WFA - yellow), astrocytic processes (AldheGFP - green), and inhibitory (vGAT - cyan) and excitatory (vGlut1 - red) synaptic terminals around cortical FSNs. Arrows point to the PNN holes containing both vGlut1 and vGAT. Bottom images represent 3D reconstruction of PNN lattice showing PNN holes containing astrocytic processes (AldheGFP-Green) as well as GABAergic (vGAT - cyan) and glutamatergic (vGlut1 - red) synaptic terminals. **j** Venn diagram showing the proportional occupancy of PNN holes by glutamatergic and GABAergic synapses with astrocytic processes. Bar diagram (bottom) shows percent of total PNN holes occupied by AldheGFP (59.41 ± 1.11), vGAT (56.74 ± 5.83), vGlut1 (70.15 ± 4.08), AldheGFP + vGAT (31.85 ± 2.73), AldheGFP + vGlut1 (46.60 ± 3.26), vGAT + vGlut1 (37.35 ± 4.71), AldheGFP + vGAT + vGlut1 (24.60 ± 3.19). 94.05 ± 1.9 % of total holes are occupied (by astrocytic markers or synapses or both) leaving 5.94 ± 1.96 % holes empty. n = >40PNNs/8s/4m. s and m represent the number of sections and mice. Bar data are expressed as mean ± SD. Scale bar 2μm in the top and ^m in bottom images in a, c, e, g, and i.

We examined >1000 PNN holes and analyzed the distribution of excitatory and inhibitory synaptic terminals in conjunction with the astrocytic marker AldheGFP on the surface of PNN-expressing cortical FSNs (Fig. 2a, b). ~80% of the PNN holes were occupied by either astrocytic processes or vGlut1 terminals or both leaving only ~20% holes unoccupied. Of all PNN holes, 71% contained excitatory synapses (vGlut1) and 53% contained astrocytic processes (AldheGFP) and 44% showed co-occupancy of excitatory synapses with astrocytic processes (Fig. 2a, b). Hence the presence of excitatory synapse in a PNN hole does not necessarily predict the presence of an astrocytic process in it; however, >60% of synaptic contacts showed the presence of astrocytic processes with them. PNN holes also contained vGlut2 expressing synapses from thalamocortical sensory projections in conjunction with astrocytic processes (Fig. S2a, b).

The inhibitory synaptic terminals in the PNN holes showed a lower astrocytic occupancy (58%), and only 33% of all PNN holes showed both astrocytic and synaptic components (Fig. 2c, d). However, similar to the vGlut1 terminals, ~58% of vGAT-occupied holes showed astrocytic contacts and overall ~80% of total PNN holes were occupied by either astrocytic processes or vGAT terminals or both (Fig. 2d).

We next asked whether astrocytic processes that colocalize with synapses in the PNN holes are equipped with transporters and Kir4.1 channels to clear synaptically released Glu, GABA, and K+.

Analysis of >1000 PNN holes showed that ~47% of all holes contained vGlut1 terminals as well as Glt1-expressing astrocytic processes (Fig. 2e, f) suggesting that ~ 75% of all excitatory terminals are accompanied by astrocytic processes equipped to uptake synaptically released Glu. On the other hand, ~35% of all holes contained both vGAT terminals and GAT3-expressing astrocytic processes thereby suggesting that ~67% of total inhibitory terminals in the PNN holes are equipped with astrocytic processes capable of GABA uptake (Fig. 2g, h). Once again ~80% of holes were occupied by one or more elements from astrocytes or synapses, or both (Fig. 2f, h) and 20% were vacant.

Interestingly vGlut1 and vGAT terminals were co-expressed in ~37% of all holes and 64% contained astrocytic processes (Fig. 2i, j). Only 33% and 19% of all holes exclusively contained vGlut1 and vGAT terminals respectively. We also observed co-expression of vGAT with vGlut2 expressing glutamatergic synapses from thalamocortical sensory projections in the PNN holes (Fig. S2c, d). Importantly, combining markers of glutamatergic and GABAergic synapses with astrocytes increased the overall occupancy of PNN holes from ~80% (Fig. 2b, d, f, h) to ~95% (Fig. 2j) suggesting that nearly all PNN holes are occupied with a mixture of synapses and astrocytic processes.

Taken together these results explicitly show that holes in the PNN contain both excitatory and inhibitory synapses and the majority of them are accompanied by astrocytic processes expressing both Glu and GABA transporters thereby suggesting the PNN holes houses a structural and functional analogue of tripartite synapses. Also, a single PNN hole can contain either excitatory or inhibitory or both types of synaptic terminals thereby ruling out the possibility of PNN holes being exclusively meant to contain a specific type of synapse or astrocytic process. Finally, our data suggest that nearly all PNN holes are filled with a synapse and astrocytic processes rarely leaving holes unoccupied.

### 3. Concurrent maturation of astrocytes with PNNs constrains the astrocytic coverage on PV neurons:

**Fig. 3:**
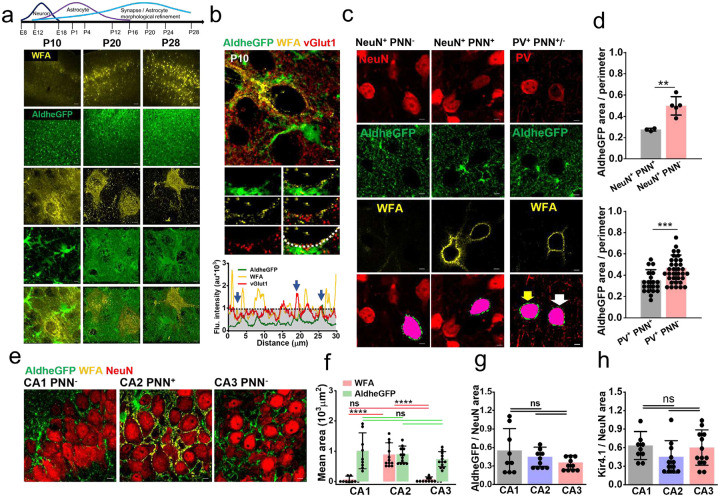
Concurrent maturation of PNN and astrocytes leads to a lower pericellular astrocytic coverage of cortical PV neurons. **a** Developmental formation and maturation of neurons, astrocytes, and synapses (top) (adapted and modified from [46]). Confocal micrographs of WFA (yellow) and AldheGFP (green) immunofluorescence in mouse cerebral cortex in postnatal days 10 (P10), 20 (P20) and 28 (P28). High magnification 3D volume images of PNNs (3^rd^ row from top) and astrocytes (4^th^ row from top) in different postnatal ages showing concurrent maturation. Scale bar 50μm in top two rows, 2μm in bottom 3 rows. **b** Top, confocal micrograph of developing PNN (WFA - yellow) showing astrocytic processes (AldheGFP - green) and excitatory synaptic terminals (vGlut1 - red). The rectangular area (white) is magnified in the bottom panel images. line intensity profile of PNN (white dotted line in bottom right image) shows peaks of vGlut1 and AldheGFP in the PNN holes (marked by arrows). Gray area represents the WFA threshold. Scale 5μm (top main image) and 2μm (bottom panel images). **c** Representative confocal micrographs showing NeuN (red), AldheGFP (green), and WFA (yellow) fluorescence in cortical neurons without (left panel) and with PNN (middle panel). Bottom images show the binarized pericellular astrocytic coverage area (green) of the cell (pink) of interest. Right panel shows confocal micrographs of PV (red), AldheGFP (green), and WFA (yellow) in the PV neurons with and without PNN. Bottom images show binarized pericellular astrocytic coverage areas (green) in PV+ neurons with (white arrow) and without (yellow arrow) a PNN. Scale 5μm. **d** Top graph showing a significantly lower pericellular AldheGFP area (normalized to cell perimeter) in NeuN PNN+ neurons (0.27 ± 0.01) compared to the NeuN+ PNN− neurons (0.49 ± 0.08). n = 22c/3m (NeuN PNN+); n = 28c/5m (NeuN PNN^−^). Bottom graph shows a significantly lower pericellular AldheGFP area (normalized to cell perimeter) of PV+ PNN+ neurons (0.35 ± 0.1) compared to the PV+ PNN^−^ neurons (0.46 ± 0.12). n = 20c/4m (PV+ PNN+); n = 34c/7m (PV+ PNN-). **e** Representative confocal micrographs showing NeuN (red), AldheGFP (green), and WFA (yellow) fluorescence in hippocampal CA1 (left), CA2 (middle), and CA3 (right) *stratum pyramidale*. Scale 5μm. **f** Bar graph representing the mean total coverage area of AldheGFP (green) and WFA (red) in CA1, CA2, and CA3 areas. Due to PNNs, WFA covered area in CA2 is significantly higher (893.23 ± 383.56) compared to CA1 (65.16 ± 112.02) and CA3 (82.03 ± 71.57); however, AldheGFP covered area in CA2 (900.02 ± 268.61) remains statistically indifferent from CA1 (1014.81 ± 594.63) and CA3 (740.86 ± 251.12). n = 9s/3m in CA1 group; 12s/4m in CA2 and CA3 groups. Red and green lines show comparisons between red (WFA) bars and green bars (AldheGFP) respectively. **g** Graphs show non-significant total astrocytic area (AldheGFP) normalized to neuronal area (NeuN) in CA1 (0.55 ± 0.35) CA2 (0.45 ± 0.15) and CA3 (0.35 ± 0.11) regions. n = 9s/3m in CA1; 10s/4m in CA2 and CA3. **h** Graphs showing statistically non-significant total astrocytic area (Kir4.1) normalized to neuronal area (NeuN) in CA1 (0.63 ± 0.22) CA2 (0.45 ± 0.26) and CA3 (0.60 ± 0.28) regions. n = 9s / 3m in CA1; 12s / 4m in CA2 and 13s/4m CA3. c, s, and m indicate the number of cells, sections, and mice respectively. Bar data are expressed as mean±SD; dots on the bars represent the individual data points. ****P < 0.0001, ***P < 0.001, **P < 0.01, *P < 0.05, ns = P > 0.05. unpaired two-tailed t-test with welch correction in d; One-way ANOVA, Tukey’s post-hoc test in f, g, and h.

PNN deposition in the developing brain coincides with the closure of the critical period of plasticity after which synapses are “locked” for future modifications provided PNNs are unaltered [17, 18, 23]. In parallel with the PNN condensation, the morphological maturation of astrocytes also occurs (Fig. 3a) [24, 25] during which astrocytic processes and synaptic contacts can both be traced to the newly formed PNN holes as evidenced in the developing brain (Fig. 3b). Based on the facts of concurrent development (Fig. 3a) and PNN holes containing both synapses and astrocytic processes (Fig. 2), we hypothesized that PNN accumulation not only locks the synapses but also stabilizes astrocytic processes at PNN holes thereby limiting the astrocytic coverage on PNN-expressing neurons only to those patches of membrane accessible through the PNN perforations.

To test this hypothesis, we compared the pericellular astrocytic coverage of PNN-expressing and non-expressing neurons identified by NeuN, AldheGFP, and WFA immunostaining (Figs. 3c, S3). Within the 0.8pm perimeter of the cell soma, PNN-expressing (NeuN+/WFA+) neurons showed a significantly lower astrocytic coverage than PNN-lacking (NeuN+/WFA−) neurons (Fig. 3c, d - top bar graph). To confirm that the lower pericellular astrocytic coverage around PV neurons is attributed to the PNN, we compared the astrocytic coverage of PNN expressing PV neurons (PV+/PNN+) with a rare cortical population of PNN lacking PV neurons (PNN−/PV+). Again, we observed a significantly higher astrocytic coverage around PNN lacking PV neurons (PV+/PNN−) than PNN expressing PV neurons (PV+/PNN−) (Fig. 3c right panel, d bottom bar graph). These data confirm that pericellular astrocytic coverage negatively correlates with the presence of the PNN most likely by restricting the access of astrocytic processes to the cell membrane to the holes within the PNN.

As mentioned above, in the hippocampus a small population of excitatory cells in CA2 express an atypical and less condensed version of PNNs[26]. Astrocytic coverage of these CA2 neurons, visualized by using astrocytic markers AldheGFP and Kir4.1, did not differ from their CA1 and CA3 counterparts lacking PNNs (Fig. 3e – h) suggesting that only the “typical” condensed PNNs on cortical PV neurons leads to restricted pericellular astrocytic coverage.

### 4. PNN disruption increases astrocytic coverage of neuronal somata:

The most straight forward interpretation of the above findings is that the highly condensed PNNs leave only a few defined sites, namely the PNN holes, for astrocytes to interact with the soma, and these holes are most likely determined by the placement of synapses during development. However, PNNs are known as dynamic structures that undergo constant homeostatic remodeling [27], which may be pivotal for allowing experience-dependent synaptic plasticity in the adult brain, and experimental degradation of PNNs has also been shown to cause synaptic plasticity[23, 28]. Hence it stands to reason that this would concomitantly change astrocytic coverage and placement of their processes.

**Fig. 4:**
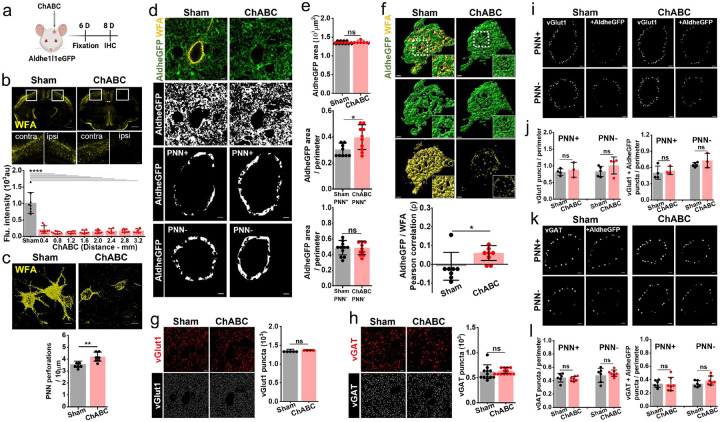
PNN disruption increases pericellular astrocytic coverage without altering synaptic contacts. **a** Schematics of intracranial ChABC injection and subsequent experiments. **b** Confocal micrographs showing immunofluorescence of WFA (yellow) in sham and ChABC injected mouse brains. Marked rectangular areas in ipsilateral (right) and contralateral (left) hemispheres are magnified in bottom panels. Scale bars, 1mm in top and 100μm in bottom images. Bar diagram of fluorescence intensity of WFA in sham and ChABC-injected groups showing widespread PNN disruption throughout the cerebral cortex. (Sham: 1019.07 ± 318.16; ChABC: 0.4mm 209.38 ± 111.13, 0.8mm 112.95 ± 37.09, 1.2mm 121.11 ± 35.20, 1.6mm 137.94 ± 52.69, 2.0mm 147.37 ± 49.09, 2.4mm 170.54 ± 65.98, 2.8mm 168.53 ± 48.71, 3.2mm 168.05 ± 61.36), n = 8s/4m in sham, 8s/8m in ChABC. **c** 3D volume images of PNNs (WFA - yellow) from sham with intact and dense PNNs compared to the ChABC injected mice showing disrupted PNNs with granulated and fragmented WFA labelling. Scale 10μm. Bar diagrams showing a significant increase in PNN perforation after ChABC-mediated PNN degradation (Sham 3.57 ± 0.25, n = 6s/4m; ChABC 4.19 ± 0.38, n = 8s/3m). **d** Confocal micrographs showing AldheGFP (green) and WFA (yellow) fluorescence (top) and AldheGFP binarized signal of total area (2^nd^ row from top), the pericellular area around PNN-expressing (3^rd^ row from top) and non-expressing (bottom) neurons in sham (left panels) and ChABC-injected (right panels) groups. **e** Bar diagram (top) of total AldheGFP area in a field of view of PNNs was unchanged in ChABC treated group (sham 1380.97 ± 23.04, n = 21i, 3m; ChABC 1393.50 ± 24.84, n = 20i, 3m). Bar diagram (middle) of normalized pericellular AldheGFP area increased significantly in PNN+ neurons in ChABC treated group (sham 0.30 ± 0.05, n = 48c/9s/7m; ChABC 0.39 ± 0.09, n = 38c/10s/7m) however remained unaltered in PNN− neurons in bottom bar diagram (sham 0.48 ± 0.09, n = 53c/11s/9m; ChABC 0.48 ± 0.86, n = 38c/10s/8m). **f** 3D reconstruction of PNN (yellow) and pericellular astrocytic coverage (green) showing increased pericellular coverage and disintegrated PNN. Inset images represent the magnified areas marked by white squares. Bar data (bottom) show an altered spatial correlation between AldheGFP and WFA in ChABC treated condition (sham −0.01 ± 0.07; ChABC 0.06 ± 0.03, n = 8c/3s/3m in each group). **g** Representative confocal images of vGlut1 fluorescence (top-red) and binary form of vGlut1 puncta (bottom-white) showing the unaltered numerical density of excitatory presynaptic puncta in ChABC treated condition (1368.63 ± 24.14, n = 55c/5s/5m) compared to sham (1374 ± 8.61, n = 21c/4s/4m). **h** Representative confocal images of vGAT fluorescence (top-red) and binary form of vGAT puncta (bottom-white) showing the unaltered numerical density of inhibitory presynaptic puncta on PNN disruption in ChABC treated condition (643.95 ± 62.87, n = 51c/14s/7m) compared to sham (620.75 ± 138.95, n = 59c/13s/5m). **i** Representative binary images from sham (left panels) and ChABC treated (right panels) groups showing pericellular vGlut1 puncta (left) and vGlut1 puncta with astrocytic contacts (right) in PNN expressing (top row) and PNN non-expressing (bottom row) cortical neurons. **j** Bar graphs showing total pericellular vGlut1 puncta (left) and vGlut1 puncta with AldheGFP contacts (right) in sham and ChABC-treated groups. The numerical density of vGlut1 puncta remained unchanged in PNN-expressing neurons (sham 0.81 ± 0.09, n = 22c/4s/4m; ChABC 0.88 ± 0.02, n = 12c/3s/3m) as well as in PNN non-expressing neurons (sham 0.83 ± 0.15, n = 27c/5s/5m; ChABC 1.01 ± 0.22, n = 11c/4s/3m). Similarly, pericellular numerical density of vGlut1 puncta with astrocytic contacts(+AldheGFP) remained unchanged in PNN expressing neurons (sham 0.50 ± 0.12, n = 22c/4s/4m; ChABC 0.54 ± 0.07, n = 12c/3s/3m) as well as in PNN non expressing neurons (sham 0.65 ± 0.03, n = 27c/5s/5m; ChABC 0.72 ± 0.13, n = 11c/3s/3m). **k** Representative binary images from sham (left panels) and ChABC treated (right panels) groups showing total pericellular vGAT puncta (left) and vGAT puncta with astrocytic contacts (right) in PNN expressing (top row) and PNN non-expressing (bottom row) cortical neurons. **l** Bar graphs showing total pericellular vGAT puncta (left) and vGAT puncta with AldheGFP contacts (right) in sham and ChABC-treated groups. The numerical density of vGAT puncta remained unchanged in PNN-expressing neurons (sham 0.44 ± 0.6, n = 26c/6s/5m; ChABC 0.42 ± 0.03, n = 24c/7s/6m) as well as in PNN non-expressing neurons (sham 0.47 ± 0.10, n = 29c/6s/5m; ChABC 0.51 ± 0.04, n = 27c/7s/6m). Similarly, pericellular numerical density of vGAT puncta with astrocytic contacts(+AldheGFP) remained unchanged in PNN expressing neurons (sham 0.33 ± 0.05, n = 26c/6s/5m; ChABC 0.33 ± 0.10, n = 24c/7s/6m) as well as in PNN non expressing neurons (sham 0.33 ± 0.04, n = 29c/6s/5m; ChABC 0.38 ± 0.06, n = 27c/7s/6m). c, s, and m indicate the number of cells, sections, and mice respectively. Bar graph data are expressed as mean±SD; dots on the bars represent the individual data points. *P < 0.05, ns = P > 0.05. unpaired two-tailed t-test with welch correction in c-I, One-way ANOVA, Tukey’s post-hoc test in b. Scale bar 5μm in field images in d, g, h; and 2μm in magnified images in d, f, i, and k.

To examine such plasticity in astrocytic coverage and synaptic contacts, we degraded cortical PNNs *in-vivo* by intracranially injecting Aldhe1l1eGFP mice with Chondroitinase ABC and compared the pericellular astrocytic coverage as well as synaptic contacts of disrupted PNNs 6-day post-injection with intact PNNs from sham (Fig. 4a). At 6-day post-ChABC injection we observed a widespread decrease in WFA intensity (Fig. 4b) as well as increased perforations (Fig. 4c) thereby making PNN less dense and more porous with an increase in extracellular space in the pericellular region.

PNN depletion did not change the total space occupied by astrocytes as determined by Aldhe1l1eGFAP in a given field of view (Fig. 4d top whole field images, e top bar diagram), however, we observed a significant increase in the cell surface-associated pericellular astrocytic coverage on PNN-expressing PV neurons (PV+PNN+) (Fig. 4d single cell binary images, e middle bar diagram) suggesting a localized change confined to the pericellular area previously occupied by the PNN. Excitatory neurons (PV^−^ PNN^−^) did not show any change in the pericellular coverage in the ChABC-treated condition (Fig. 4d bottom single cell binary images, e bottom bar diagram) suggesting that the increased astrocytic coverage is indeed associated with the depletion of the PNN but not diffuse CSPG. The increased astrocytic coverage also changed the non-overlapping spatial arrangement of the astrocytic processes with the remnant of the PNNs as observed in the positive spatial correlation between astrocyte and WFA (Fig. 4f).

We next examined whether the increased pericellular astrocytic coverage following ChABC treatment also increases the number of astrocytic contacts in newly created holes in a now highly perforated PNN (Fig. 4c). Indeed, using a combination of astrocytic markers Glt1 and AldheGFP, we observed a significantly higher number of PNN holes that were now occupied with astrocytic processes (Fig. S4a – c). In control mice, we observed ~60–70% of the PNN holes being occupied by at least one of the astrocytic markers (Figs. 1, S4b, c), whereas, in the ChABC-treated group, this number increased to 80–90% (Fig. S4b, c).

PNNs are known to stabilize synapses and depletion of PNNs is known to alter axosomatic synaptic contacts in several brain areas[28, 29]. Since astrocytes seem to be an integral component of the axosomatic synapses in the PNN holes, we next asked whether the observed changes in pericellular astrocytic coverage resulting from PNN depletion also alter or destabilize the axosomatic synaptic contacts. To this end, we first compared the total number of vGlut1 and vGAT expressing synaptic terminals; surprisingly ChABC treatment neither changed the total vGlut1 (Fig. 4g) nor the total vGAT terminals (Fig. 4h). Next, we restricted our analysis to the pericellular area in which astrocytic coverage was increased; here too no significant change in the numerical densities of pericellular vGlut1 (Fig. 4i, j) and vGAT (Fig. 4k, l) contacts were found upon ChABC treatment in either PNN expressing or non-expressing neurons. We also assessed the pericellular vGlut1 (Fig. 4i, j) or vGAT (Fig. 4k, l) contacts closely associated with the astrocytic processes however no significant changes were observed in these groups too.

Finally, we analyzed whether following ChABC treatment PNN holes show any changes in their occupancy of the synaptic terminals. Using the line intensity profile method, we assessed the occupancy of PNN holes in the ChABC-treated group. Despite a significantly higher occupancy of PNN holes by astrocytic processes (Fig. S4b, c), no significant changes were observed either in the occupancy of vGlut1+ terminals (Fig. S4d, e) or vGlut1+ terminals with astrocytic contacts (Fig. S4f) within PNN perforations. Similarly, no significant differences were found in total vGAT+ terminals (Fig. S4g, h) as well as in vGAT+ terminals in close association with astrocytes (Fig. S4i) within PNN perforations.

Taken together these studies suggest that axosomatic synapses embedded in PNN holes are highly stable and resistant to degradation of the PNNs in the adult somatosensory cortex; however astrocytic processes in the same compartment are highly plastic and undergo structural changes independent of synaptic terminals. It, therefore, appears as if astrocytes tend to occupy as much of the free neuronal surface as is accessible.

### 5. Permanent PNN depletion induces astrocytic plasticity without altering synaptic stability:

Since temporary degradation of PNNs using ChABC changed astrocytic coverage by occupying newly created perforations without changing the overall pericellular numerical abundance of presynaptic terminals or synaptic terminals with astrocytic contacts on PV neurons, we sought to investigate whether a permanent genetic deletion of PNNs destabilizes the axosomatic synapses in conjunction with changing the pericellular astrocytic coverage.

**Fig. 5:**
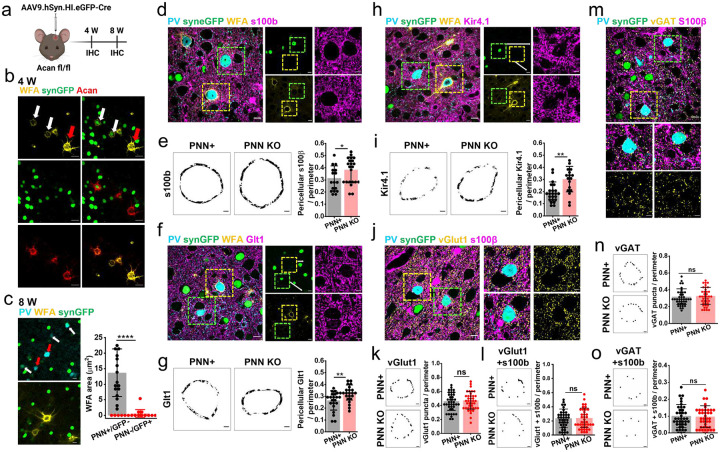
AAV-mediated permanent PNN disruption increases pericellular astrocytic coverage without altering synaptic contacts. **a** Schematics of AAV9.hSyn.HI.eGFP-Cre mediated PNN knockout in adult Acan fl/fl mice. **b - c** Confocal micrographs of PV (cyan), WFA (yellow), synGFP (green), and aggrecan (red) immunofluorescence from the cortical section of acan fl/fl mice showing (**b**) partial PNN disruption after 4 weeks and (**c**) complete disruption after 8 weeks of AAV9.hSyn.HI.eGFP-Cre injection. AAV-infected and non-infected cells are marked by white and red arrows respectively. Bar graph in c showing a negligible WFA area in AAV9.hSyn.HI.eGFP-Cre infected (PNN-/GFP+) cells compared to the non-infected cells (PNN+/GFP-). Control 13.66 ± 7.65, n = 17 i/3m; ChABC 0.61 ± 1.21, n = 21i/3m. Scale 20Mm in b and 10Mm in c. **d** Confocal micrograph of PV (cyan), SynCreGFP (green), PNN (WFA - yellow), and astrocyte (s100b - magenta) fluorescence in Acan fl/fl injected with SynCreGFP prefrontal cortical section. Transduced PV neurons (green square) show PNN elimination compared to the non-transduced PV neurons with intact PNNs (yellow square). The left panels show magnified areas in the squares. **e** Binary images of pericellular s100b area from PV neuron with intact PNN (PNN+, left) and AAV-mediated PNN KO (right). Bar graph (right) shows increased pericellular s100b coverage on PV neurons with PNN KO (PNN+ 0.31 ± 0.10, n = 17c/3m; PNN KO 0.38 ± 0.10, n = 22c/3m). **f** Confocal micrographs of PV (cyan), SynCreGFP (green), PNN (WFA - yellow), and astrocyte (Glt1 - magenta) fluorescence in Acan fl/fl injected with SynCreGFP prefrontal cortical section. Transduced PV neurons (green square) show PNN elimination compared to the non-transduced PV neurons with intact PNNs (yellow square). Right panels show magnified areas in the squares. **g** Binary images of pericellular Glt1 area from PV neuron with intact PNN (PNN+, left) and AAV-mediated PNN KO (right). Bar diagram (right) shows increased pericellular Glt1 coverage on PV neurons with PNN KO (PNN+ 0.26 ± 0.08, n = 20c/3m; PNN KO 0.33 ± 0.06, n = 26c/3m). **h** Confocal micrographs of PV (cyan), SynCreGFP (green), PNN (WFA - yellow), and astrocyte (Kir4.1 - magenta) fluorescence in Acan fl/fl injected with SynCreGFP prefrontal cortical section. Transduced PV neurons (green square) show PNN elimination compared to the non-transduced PV neurons with intact PNNs (yellow square). Right panels show magnified areas in the squares. **i** Binary images of pericellular Kir4.1 area from PV neuron with intact PNN (PNN+, left) and AAV-mediated PNN KO (right). Bar diagram (right) shows increased pericellular Kir4.1 coverage on PV neurons with PNN KO (PNN+ 0.19 ± 0.08, n = 23c/3m; PNN KO 0.30 ± 0.10, n = 17c/3m). **j** Confocal micrographs of PV (cyan), SynCreGFP (green), glutamatergic synapses (vGlut1 - yellow), and astrocyte (s100b - magenta) fluorescence in Acan fl/fl injected with SynCreGFP prefrontal cortical section. Yellow and green squares mark the magnified areas in top and bottom panels showing non-transduced and transduced PV neurons respectively. **k** Binary images of pericellular vGlut1 puncta on PV neuron with intact PNN (PNN+, top) and AAV-mediated PNN KO (bottom). Bar diagram (right) shows unaltered pericellular vGlut1 puncta on PV neurons with PNN KO (PNN+ 0.46 ± 0.12, n = 40c/4m; PNN KO 0.47 ± 0.12, n = 40c/4m). **l** Binary images of pericellular vGlut1 puncta with s100b processes from PV neuron with intact PNN (PNN+, top) and AAV-mediated PNN KO (bottom). Bar diagram (right) shows unaltered pericellular vGlut1 puncta with s100b processes on PV neurons with PNN KO (PNN+ 0.23 ± 0.13, n = 40c/4m; PNN KO 0.24 ± 0.13, n = 40c/4m). **m** Confocal micrographs of PV (cyan), SynCreGFP (green), GABAergic synapses (vGAT - yellow), and astrocyte (s100b - magenta) fluorescence in Acan fl/fl injected with SynCreGFP prefrontal cortical section. Yellow and green squares mark the magnified areas in the bottom panels showing non-transduced and transduced PV neurons respectively. **n** Binary images of pericellular vGAT puncta on PV neuron with intact PNN (PNN+, top) and AAV-mediated PNN KO (bottom). Bar diagram (right) shows unaltered pericellular vGAT puncta on PV neurons with PNN KO (PNN+ 0.31 ± 0.09, n = 40c/4m; PNN KO 0.32 ± 0.10, n = 40c/4m). **o** Binary images of pericellular vGAT puncta with s100b processes from PV neuron with intact PNN (PNN+, top) and AAV-mediated PNN KO (bottom). Bar diagram (right) shows unaltered pericellular vGAT puncta with s100b processes on PV neurons with PNN KO (PNN+ 0.10 ± 0.06, n = 40c/4m; PNN KO 0.09 ± 0.06, n = 40c/4m). c, i, and m indicate the number of cells, images, and mice respectively. ****P < 0.0001, **P < 0.01, *P < 0.05, ns = P > 0.05 unpaired two-tailed t-test with Welch correction. Bar data are expressed as mean±SD; dots on the bars represent the individual data points. Scale bar d - o: 10Mm large images, 5Mm magnified images, 2Mm binary images.

We disrupted PNNs permanently by intracranially injecting a viral vector carrying Cre recombinase with eGFP reporter AAV9.hSyn.HI.eGFP.WPRE.SV40 (AAV9.Cre) in the prefrontal cortex of adult Acan fl/fl mice (Fig. 5a) as described previously[27]. Consistent with the previous studies[27], PNNs show partial degradation at 4 weeks (Fig. 5b) followed by a complete elimination after 8 weeks (Fig. 5c) of AAV9Cre injection in all transduced PV neurons. Since Acan fl/fl mice lack genetic labelling of astrocytes with eGFP (AldheGFP), we used 3 different astrocytic markers S100b, Glt1, and Kir4.1 in conjunction with PV and WFA to quantify the pericellular coverage of astrocytes upon permanent PNN depletion. We compared the pericellular astrocytic coverage and numerical density of synapses of AAV9Cre-transduced PV neurons showing PNN KO with non-transduced PV neurons showing intact PNN (Fig. 5d, f, h) present in the close vicinity.

With all three markers including S100β (Fig. 5d, e), Glt1 (Fig. 5f, g), and Kir4.1 (Fig. 5h, i), the pericellular astrocytic coverage showed a consistent increase around the PV neurons with PNN deletion compared to their control counterparts with intact PNNs suggesting that once changed, astrocytes maintain the coverage in the absence of the PNNs. However, to our surprise, we did not observe any changes in either the pericellular density of all vGlut1 terminals (Fig. 5j, k) or vGlut1 terminals associated with s100b-expressing astrocytic processes (Fig. 5l). Similarly pericellular density of all vGAT-labelled inhibitory synaptic terminals (Fig. 5m, n) or vGAT terminals associated with astrocytic processes (Fig. 5o) remained unaltered on PNN elimination around PV neurons.

These data suggest that astrocytic processes undergo localized structural changes upon PNN elimination leading to an increase in the astrocytic coverage around the PV neurons. A temporary degradation of PNNs is sufficient to induce this astrocyte plasticity. Permanent and selective PNN elimination induce similar astrocytic structural plasticity around PV neurons however in both cases pericellular abundance of excitatory and inhibitory axosomatic synapses with and without astrocytic contacts remained unaltered.

### 6. PNN facilitates astrocytic uptake of synaptically released Glu:

Since astrocytic processes tightly associate with the axosomatic tripartite synapses embedded in the PNNs, it is possible that PNNs may also participate functionally in neurotransmitter and ion homeostatic functions of astrocytes. Specifically, we hypothesize that the negatively charged CSPGs associated with PNNs may electrostatically interact and aid to contain synaptic activity-released charged ions and neurotransmitters, particularly Glu and K+. This may be of particular importance in fast-spiking PNN expressing PV interneurons.

If true, we predict that disruption of PNN assembly should reduce astrocytic Glu uptake upon synaptic activity. Since this hypothesis solely relies on the physical assembly of the PNN, an acute depletion of PNN should be disruptive to astrocytic Glu uptake without any long-term changes in the expression of Glu transporter expression in astrocytic processes.

Incubation of brain slices with ChABC completely dissolved PNNs (Fig. S5a) without altering biophysical properties of patch-clamped astrocytes compared to undigested control slices each showing characteristic negative and stable resting membrane potential (Fig. S5b, c), and low membrane capacitance (Fig. S5d) as well as input resistance (Fig. S5e). Also, input-output curves were similar in control and ChABC-treated slices (Fig. S5f, g).

**Fig. 6:**
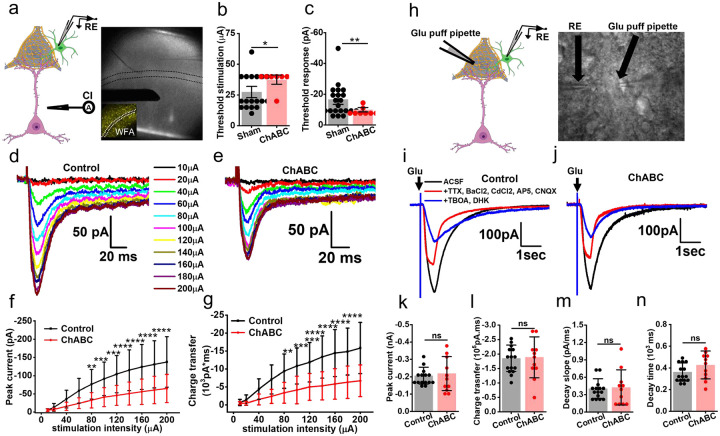
Acute PNN depletion disrupts glutamate uptake by astrocytes. **a** Schematics showing stimulation of glutamatergic neurons by a current injector (CI) causing glutamate release by synapses and subsequent uptake by astrocytic processes in the PNN holes. Brightfield image showing placement of stimulator (CI) and recording electrode (RE) in the cortical slice. The Inset image shows WFA labelling in a cortical slice. **b - c** Bar diagrams showing significantly higher threshold stimulation (**b**) (control 27.5 ± 13.52, n = 20c/10m; ChABC 37.5 ± 7.07, units mA, n = 8c/5m) and lower glutamate uptake threshold response (**c**) (control −16.6 ± 10.07, n = 22c/10m; ChABC −9.9 ± 2.5, n = 8c/5m, units pA) in ChABC treated slices than control slices. **d - e** Representative glutamate uptake current traces recorded from cortical astrocytes in response to a series of incrementing field stimulate in control (**d**), and in ChABC treated (**e**) acute slices. **f - g** Input-output curves recorded from cortical astrocytes showing significantly lower peak glutamate uptake currents (**e**), as well as a lower charge transfer (**f**) in ChABC-treated slices compared to control. n = 24c/10m; ChABC 16c/6m both **f** and **g**. **h** Schematics of recording method (left) and bright-field image (right) of an acute slice showing the recording electrode (RE) and glutamate puff electrode. **i - j** Representative voltage clamp traces of astrocytic currents on puffing 200μM glutamate in presence of various blockers to isolate glutamate uptake current in control (**i**) and ChABC-treated (**j**) acute cortical slices. **k - n** Bar diagrams showing unchanged (**k**) peak uptake current (control −206.51 ± 48.59; ChABC −218.77 ± 97.48), (**l**) total charge transfer (control −154042.79 ± 45650.84; ChABC −188759.21 ± 70795.66), (**m**) decay slope (control 0.405 ± 0.17; ChABC 0.425 ± 0.30), and (**n**) current decay time (control 362.80 ± 83.68; ChABC 427.35 ± 128.51) of astrocytes in ChABC treated slices compared to control slices. n = 15c/8m in the control and 11c/5m in ChBAC treated group. Units, pA (c), pA.ms (d), pA/ms (e), and ms (e). c and m indicate the number of cells and mice respectively. ****P < 0.0001, ***P < 0.001, **P < 0.01, *P < 0.05, ns = P > 0.05. unpaired two-tailed student t-test (equal variance not assumed) in b, c, j-m; two-way ANOVA, Tukey’s post-hoc test in f and g. Bar data are expressed as mean±SD; dots on the bars represent the individual data points.

To test the hypothesis that PNNs affect the clearance of synaptically released Glu, we stimulated L5–6 axonal fibers and recorded from astrocytes in L3–4, wherein a high density of PNNs makes it possible for each astrocyte to contact multiple PNNs (Fig. 6a). The distance between the stimulator and patch pipette was kept identical for all experiments. Synaptically-evoked glutamate currents were recorded in the presence of Bicuculline, BaCl2, AP-5, and CNQX as described previously[30–32]. Astrocytic Glu transporters current was blocked with a cocktail of TBOA and DHK, with the small fraction of remaining current being completely blocked by 0.5Mm tetrodotoxin confirming that the recorded current was indeed the synaptically evoked glutamate uptake current (Fig. S5h).

In the first set of experiments, we determined the minimum amount of current (threshold stimulation) required to generate a reliably detectable threshold response. In ChABC-treated slices, astrocytic responses to synaptically released glutamate required significantly higher current injections than controls (Fig. 6b) and still generated significantly smaller threshold responses (Fig. 6c) compared to controls.

In the next set of experiments, we generated input-output curves of astrocytic glutamate uptake currents. After assessing multiple ranges of stimuli, we observed a near linear range with 10–200mA stimulation with a 20mA increment suitable for the input-output curve. ChABC-treated slices showed a significant decrease in the peak glutamate uptake (Fig. 6d – f) as well as reduced total charge transfers (Fig. 6g) suggesting that the presence of intact PNNs yields an increase in astrocytic glutamate uptake in response to synaptic activation.

To ensure that the reduced Glu uptake is not due to a non-specific loss of astrocytic Glt-1 transporter expression of function, we repeated these experiments supplying exogenous glutamate pulses from a set distance to astrocytes while blocking synaptic transmissions and other nonspecific currents as described previously [33, 34] (Fig. 6h). Under these conditions, astrocytes showed no significant change in the glutamate uptake current (Fig. 6i–k) or uptake kinetic (Fig. 6l, m) after ChABC digestion. Furthermore, immunohistochemical analysis of Glt-1 expression as well as AldheGFP membrane area remained unaltered after ChABC digestion (Fig. S6a–e) suggesting that altered glutamate uptake by astrocytes was not attributed to a change in the glutamate transporter expression but more likely to a spillage of Glu out of the synapse and out of the reach of the patch-clamped astrocyte.

### 7. PNN facilitates astrocytic uptake of depolarization released K^+^:

The above data suggest that PNN holes that harbor synapses and astrocytic processes act as a container to prevent synaptically released glutamate to spill from the synaptic cleft. Fast-spiking interneurons also release copious amounts of K^+^. Hence, we questioned whether PNN may also aid in containing K^+^ diffusion into the perisynaptic space.

**Fig. 7:**
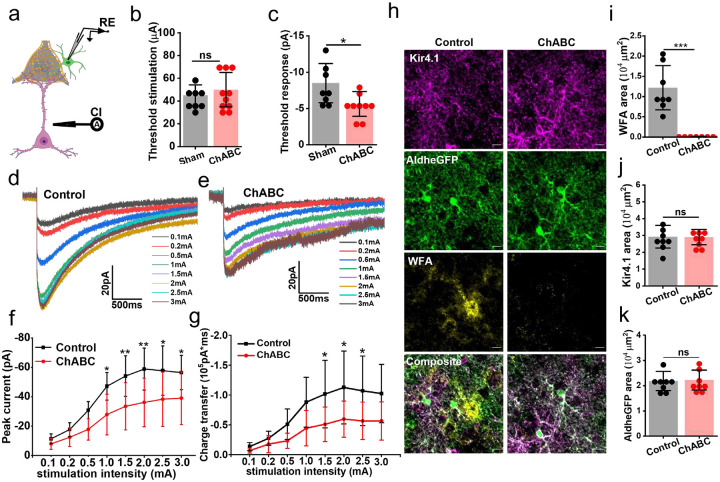
Acute PNN digestion disrupts potassium uptake by astrocytes. **a** Schematics showing stimulation of glutamatergic neuron by a current injector (CI) causing neuronal depolarization and release of extracellular potassium and subsequent uptake by astrocytic processes. **b-c** Bar diagrams showing (**b**) threshold stimulation (control: 45 ± 9.25μA, n = 8c/3m; ChABC: 47.77 ± 14.16 μA, 10c/5m) and (c) significantly lower threshold K+ current response (control: −8.62 ± 2.69pA, n = 8c/3m; ChABC: −5.627 ± 1.70pA, n = 9c/5m) in ChABC treated slices than control slices. **d - e** Representative K^+^ uptake current traces recorded from cortical astrocytes in response to a series of incrementing field stimuli in control (**d**) and ChABC-treated (**e**) acute slices. **f - g** Input-output curves recorded from cortical astrocytes showing (**f**) significantly lower peak K+ uptake currents, as well as (**g**) a lower charge transfer in ChABC-treated slices compared to control. n = control 11c/7m; ChABC 15c/6m in **f**; and n = control 11c/7m; ChABC 13c/6m in g. ***P < 0.001, **P < 0.01, *P < 0.05, ns = P > 0.05. Two-way ANOVA, Tukey’s post-hoc test. **h** Confocal micrographs of Kir4.1 (magenta), aldheGFP (green), and WFA (yellow) from fixed acute slices from control and after ChABC treatment. Scale bar 10μm. **i - k** Bar diagrams of immunofluorescence area of WFA (i) (control 12175.74 ± 5481.42, ChABC 84.24 ± 40.79 μm^2^), Kir4.1 (j) (control 29277.79 ± 6735.50, ChABC 29123.26 ± 4496.46 μm^2^), and AldheGFP (k) (control 21833.27 ± 3826.81, ChABC 22221.49 ± 3948.68μm^2^) showing PNN disruption without any changes in astrocytic Kir4.1 expression. N = 8s/4m in each group. c, s, and m indicate the number of cells, slices, and mice respectively. ****P < 0.0001, ***P < 0.001, **P < 0.01, *P < 0.05, ns = P > 0.05. unpaired two-tailed student t-test with Welch correction in b, c, I, j, and k. Two-way ANOVA with Tukey’s post-hoc test in f and g. Bar data are expressed as mean±SD; dots on the bars represent the individual data points.

To this end, we recorded L5–6 induced depolarization K^+^ currents in L3–4 astrocytes in the presence of a cocktail blocking postsynaptic and astrocytic glutamate currents comparing control and ChABC-treated slices (Fig. 7a). As was the case with Glu uptake, astrocytic K^+^ uptake was also significantly attenuated on PNN disruption. Although astrocytes required similar magnitudes of threshold stimuli (Fig. 7b) to evoke a detectable K^+^ current, the threshold response was significantly lower in the ChABC-treated slices (Fig. 7c). Complementing the threshold response current, the input-output curve of the synaptically evoked K^+^ currents showed a significantly lower K^+^ uptake current (Fig. 7d, e, f) resulting in a reduced total charge transfer (Fig. 7d, e, g). To eliminate the possibility of altered expression of Kir4.1 contributing to the lower K^+^ uptake by astrocytes, we performed immunohistochemical analysis of Kir4.1 in recorded slices. ChABC treatment eliminated PNNs as seen in significantly lower WFA reactivity (Fig. 7h, i), however Kir4.1 (Fig. 7h, j) AldheGFP (Fig. 7h, k) expressions were unaltered in ChABC-treated slices suggesting that altered K^+^ currents could not be attributed to a change in Kir4.1 expression in astrocytes.

Taken together this data suggests that intact PNNs ensure the effective uptake of K^+^ and Glu into astrocytes, and PNN disruption causes reduced uptake and accumulation of extrasynaptic Glu most likely as synaptically released molecules can now diffuse into the extrasynaptic space.

## Discussion:

PNNs have fascinated neuroscientists since their description by Golgi over a century ago. Their role in stabilizing synapses has been well documented in the visual system where PNN disruption can re-establish synaptic plasticity. Those studies, however, were unaware that most excitatory and many inhibitory synapses are ensheathed by astrocytic processes or leaflets and together form the tripartite synapse. Here astrocytes are well positioned to support synaptic function through the effective clearance of neuronally released transmitters and ions. The major objective of our study was to examine the hypothesis that PNNs may be an important structural and functional component at the tripartite synapse.

To do so we elected to focus our work on cortical fast-spiking PV+ interneurons, as 90% of them express PNNs. Our studies in adult animals shed light on a number of structural and functional roles of PNNs that have not been recognized before. Notably, to the best of our knowledge, ours is the first study to describe the functional cooperation of PNNs and astrocytes in the clearance of neuronally released Glu and K^+^ at tripartite synapses. Firstly, we show that about 90% of PNN holes contain either excitatory synapses, inhibitory synapses, or both. Secondly, about 70% of these synapses are tripartite, hence contain astrocytic processes or leaflets. In all instances, these express Kir4.1 the astrocytic channel tasked with K^+^ uptake as well as Glt-1, the major excitatory amino acid transporter in astrocytes irrespective of whether they are associated with an excitatory or inhibitory synapse. Interestingly they do not harbor aquaporins, the water channels abundantly expressed in conjunction with Kir4.1 channels on astrocytic endfeet touching blood vessels. Thirdly we used transient enzymatic or permanent genetic disruption of the PNNs to ascertain whether the astrocytic investment in tripartite synapses on inhibitory neurons is stable or plastic, and to ask whether the synapse itself is static or shows plasticity when PNNs are lost. To our surprise, we found that in the adult cortex, synapses on inhibitory neurons are static and do not change with PNN removal. However, astrocytic processes are plastic and upon PNN removal astrocytes expand their territory on the cell soma. It almost appears as if astrocytes take possession of any membrane that was previously occupied by PNNs. Interestingly, this picture appears different from the developing cortex where PNN degradation has been shown to reestablish synaptic plasticity in the visual system[35].

**Figure 8:**
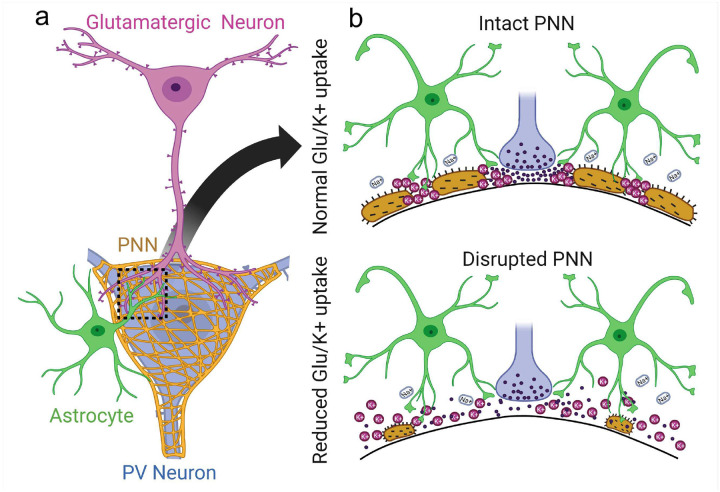
PNN facilitates astrocytic uptake of synaptic activity released glutamate and potassium. **a** PNN-enclosed PV neurons receive glutamatergic inputs in the PNN holes wherein astrocytic processes are also confined (magnified area) and make this space analogous to the tripartite synapse. **b** Under physiological conditions, when PNN is intact (top), glutamate released during synaptic activity and extracellular potassium ions released upon neuronal repolarization are directed towards astrocytic processes by the PNN to aid efficient clearance of extracellular glutamate and potassium. When PNNs are disrupted (bottom), glutamate and potassium ions escape from and diffused laterally into the extracellular space.

While the structural interactions of synapses and astrocytes, and the structural astrocyte plasticity are fascinating, we also uncovered an important functional synergism between PNNs and astrocytes. Having the synapse embedded in negatively charged ECM constituents, the PNN walls off the synapse prevent lateral diffusion of ions and neurotransmitters, and contain them in the local pocket or cavity where astrocytic leaflets can effectively remove all the synaptically released transmitter as well as K^+^ released in the process (Fig. 8). Not so when the PNNs are disrupted and Glu spills out of the synapses as evident from increased [Glu] in the perisynaptic space. As PNNs are known to be degraded by proteolytic enzymes including MMPs and ADAMTSs [6, 36, 37], it is easy to envision how brain inflammation in the context of injury or disease will break down PNNs and hence allow spillage of K^+^ and Glu into the perisynaptic space. Increases in K^+^ and Glu are known contributors to epilepsy, and at least in glioma-associated epilepsy, it has been demonstrated that inhibition of PNN proteolysis suppresses seizures[22]. Furthermore, it is well documented that activation of extrasynaptic NMDA-R by spillage of Glu can induce neuronal death via activation of the p38MAPK pathway[4]. Hence, we propose that the containment of axosomatic synapses on PV interneurons is a protective strategy. This may be particularly important for this group of neurons, which are among the fastest-firing neurons in the brain. Reported to be capable of firing 100s of action potentials per second, the release of K^+^ by these neurons will far exceed that of most normal spiking neurons in the brain. The negative charges on the walls of the PNN holes may temporarily bind the positively charged K^+^ ions thereby neutralizing them temporarily allowing astrocytes additional time to buffer K^+^ during following bursts of neuronal activity without risking depolarization of the membrane. This is not far-fetched in light of the previously reported charge density of PNNs estimated at over 100 mM[38]. Hence PNNs may be a structure that evolved specifically to endow fast-spiking neurons to handle the shift in ions and charged neurotransmitters that are associated with rapid firing. We recently showed that PNNs around cortical PV neurons also change the dielectric properties of the cell membrane such as to reduce the effective membrane capacitance. This facilitates rapid burst firing of the neurons and we show in a glioma mouse model that enzymatic degradation of PNNs, by MMPs released from the tumor degrades PNNs, increases the membrane capacitance, and impairs fast burst firing[22].

The finding that PNN degradation impairs Glu uptake and K+ buffering by astrocytes at tripartite synapses may have general applicability relevant to numerous acute and chronic neurological conditions. This may obviously contribute to the generation of seizures which are known to cause the release of matrix metalloproteinases (MMPs) [39, 40]. Moreover, changes in PV interneuron firing have been reported in the PFC in the context of Schizophrenia [41], and in the substantia nigra the release of dopamine comes from fast-spiking interneurons that may be negatively affected in Parkinson disease [42, 43]. It is possible that in these diseases and others, loss of PNN integrity may also affect the astrocytes’ ability to support effective clearance of neuronally released K^+^ and Glu. The idea that synaptic function and not just structure critically depends on functional contribution of astrocytes working synergistically with the PNN, dubbed the “tetrapartite synapse” [44, 45], is an exciting concept that clearly warrants further study.

## Materials and methods:

### Mice:

All animal procedures were approved and performed following the ethical guidelines set by the University of Virginia Institutional Animal Care and Use Committee (IACUC) ACUC. Mice were housed in groups of five in a facility in 12 h light/dark cycles and had access to food and water *ad libitum*. AldheGFP mice expressing enhanced green fluorescence protein (eGFP) under astrocyte-specific promotor AldheGFP were generated as described previously [33, 34] and housed and bred according to ACUC guidelines. We received C57BL/6N-Acan<tm1a(EUCOMM)Hmgu>/H mice (common name HEPD0602_5_G11) from EUCOMM (UK Research & Innovation, Mary Lyon Center, Harwell Campus, strain ID # EM:10224). The heterozygous mice were bred together to generate Acan fl/fl mice and confirmed by genotyping before being used for any experimentation.

### Intracranial surgeries and injections:

#### ChABC injection

Chondroitinase ABC from *Proteus vulgaris* (cat # C3667–10UN, Sigma Aldrich) was dissolved in sterilized PBS (50MU/μl); subsequently, 2 μl solution was injected unilaterally at an infusion rate of 200nl/minute. Mice were anesthetized with 2–5% isoflurane and fixed to a stereotaxic apparatus (Leica Angleone stereotaxic model 39464710) followed by a midline scalp incision and a 0.5 mm burr hole 2.0mm lateral and 1.0mm ventral to bregma and infused ChABC at ~2.0mm deep from the cortical surface using a 10μl syringe (World Precision Instruments #SGE010RNS). Sham control mice were injected with sterile PBS with an identical procedure. Mice were dosed with Buprenorphine / Rimadyl and allowed to recover on a heating pad until mobile and were monitored daily for up to 5 days from surgery. Body weight was measured for 3 consecutive days after surgery and all mice were perfused on 6^th^-day post-injection.

#### AA V injection and Acan knockout:

To achieve PNN knockout in adult mice brains, we injected pENN.AAV.hSyn.HI.eGFP-Cre.WPRE.SV40 (Addgene #105540-AAV9) in 7–8 weeks old Acan fl/fl mice. In brief AAV9 (2.7x 10^13^ vg/mL) was diluted in sterile PBS to achieve 1x 10^13^ vg/mL concentration and 1.5μl was injected in each hemisphere (from bregma: 0.5mm posterior, 2.0mm lateral, 1.0mm ventral) with 200nl/minute infusion rate as described above. Mice were transcardially perfused after 8–10 weeks of AAV9 injections to perform IHC.

### Acute slice electrophysiology:

Whole-cell patch-clamp recordings were obtained from astrocytes *in situ* acute brain slices as described previously[30, 31, 34]. In brief, mice underwent cervical dislocation followed by a quick decapitation and dissection to remove brains and were kept in an ice-cold ACSF (135mM NMDG, 1.5mM KCl, 1.5mM KH2PO4, 23mM choline bicarbonate, 25mM D glucose, 0.5mM CaCl2, 3.5mM MgSO4; pH 7.35, 310 ± 5 mOsm) (All from Sigma-Aldrich) saturated with carbogen (95% O2 + 5% CO2). We prepared 300 μm thick coronal slices using Leica VT 1000P or 1200S tissue slicers. Slices were transferred into a custom-made recovery chamber filled with ACSF (125mM NaCl, 3mM KCl, 1.25mM NaH2PO4, 25mM NaHCO3, 2mM CaCl2, 1.3mM MgSO4, 25mM glucose, pH 7.35, 310 ± 5 mOsm) constantly bubbled with carbogen (95% CO2 + 5% O2) to recover at 32 °C for 1 hr. Subsequently, slices were transferred to room temperature conditions until used for recordings. Individual slices were transferred to a recording chamber that was continuously superfused with ACSF at a flow rate of 2 ml/min. GFP-positive astrocytes in Aldh1l1-eGFP mice cortical slices were visualized using an upright microscope (Leica DMLFSA) with 5x and 40x water immersion lens and epifluorescence and infrared illuminations to identify eGFP-expressing astrocytes.

Whole-cell voltage-clamp and current-clamp recordings were achieved using an Axopatch 200B amplifier (Molecular Devices) with an Axon Digidata 1550A interface (molecular devices). Patch pipettes of 7–10MΩ open-tip resistance were created from standard borosilicate capillaries (WPI, 4IN THINWALL Gl 1.5OD/1.12ID) using HEKA PIP 6 (HEKA) or PMP-102 (Warner instruments) programmable pipette pullers. We filled patch pipettes with an intracellular solution containing 134mM potassium gluconate, 1mM KCl, 10mM 4-(2-hydroxyethyl)- 1-piperazineethanesulfonic acid (HEPES), 2mM adenosine 5’-triphosphate magnesium salt (Mg-ATP), 0.2mM guanosine 5’-triphosphate sodium salt (Na-GTP) and 0.5mM ethylene glycol tetraacetic acid (EGTA) (pH 7.4, 290–295 mOsm). MM-225 micromanipulator (Sutter Instrument, Navato, CA) was used to visually guide the patch pipette to the cell. After making a tight seal of >5GΩ resistance, brief suction was applied to achieve the whole-cell mode and cells were immediately clamped at −80mV. The membrane capacitance (Cm) and series resistance were not compensated unless otherwise stated. Data were acquired using Clampex 10.4 software and Axon Digidata 1550A interface (Molecular Devices), filtered at 5 kHz, digitized at 10–20 kHz, and analyzed using Clampfit 10.6 or Clampfit 11.2 software (Molecular Devices). Unless otherwise stated, throughout all the recordings carbogen-bubbled ACSF was continuously superfused (2 ml/min) and the bath temperature inside the reordering chamber was maintained at 32–33 °C using an inline feedback heating system (Cat# TC 324B, Warner Instruments).

### PNN degradation in ex-situ brain slices:

Chondroitinase ABC (ChABC) from *Proteus vulgaris* (Cat# C3667, Sigma-Aldrich) was reconstituted in a 0.01% bovine serum albumin aqueous solution according to the manufacturer’s instruction to make a 1 U/40 μl stock solution. Aliquots of 1U were prepared and stored at −20 °C until used. After slice recovery slices were treated with ChABC and subsequent recordings were made as previously described[21, 22]. In brief, after recovery, 2–3 cortical half slices were incubated in 3ml ChABC solution (0.5 U ChABC / ml ACSF) in an incubation chamber continuously supplied with carbogen at 33 °C for 45 min. Next, the slices were rinsed twice and incubated in ACSF until used for electrophysiological recordings. These parameters of PNNs digestion by ChABC (enzyme concentration—0.5 U/ml, incubation time - 45 min, incubation temperature - 33 °C) reliably degraded PNNs (Fig. S5a) as described previously[21, 22]. For controls, previously separated contralateral halves of the ChABC-treated slices were incubated in 3ml of ACSF without ChABC, and subsequently, both ChABC-treated and non-treated slices were kept in ACSF together until used for the recordings.

### Measurement of intrinsic biophysical properties of astrocytes:

The resting membrane potential (Vm) was measured by setting I = 0 mode immediately after achieving the whole-cell configuration. Membrane capacitance (Cm) was measured directly from the amplifier by adjusting capacitance and monitoring the capacitive transients as described previously[30]. To calculate the input resistance (Rin) of astrocytes, we calculated the steady-stated membrane voltage deflection (ΔV) on injecting 15 hyperpolarization current steps (-100 pA each for 1000 ms). The ratio (ΔV/l) of steady-state change in the membrane voltage (ΔV) and the corresponding injected current (I) was computed as Rin. The I-V curve of astrocytes was computed in both the current clamp (31 steps, −100pA to +500pA, step size 20pA, step duration 1100ms) and voltage clamp (25 sweeps, −180mV to +60mV, step size 10mV) modes (Fig. S5f – i) followed by plotting the voltage/current responses. Astrocytes with nonlinear IV responses were not continued for recordings and analysis.

### Measurement of astrocytic currents:

#### Synaptically evoked Glutamate uptake current:

We recorded synaptically evoked currents from cortical astrocytes according to the previously published studies with some modifications[31, 47]. In brief, we placed a concentric bipolar electrode (FHC, # CBABD75) in L5–6 of the prefrontal cortical slices and patched astrocytes in L 3–4 (Fig. 6a). The stimulation protocol consists of initial 10μA and 20μA pulses followed by a 20μA increment in each subsequent pulse capping at 200μA (pulse duration 200μs). All recordings were performed in presence of 20μM bicuculline, 50 μM D-2-amino-5-phosphonovalerate (D-AP-5), 20 μM 6-cyano-7-nitroquinoxaline-2,3-dione (CNQX), and 100μM BaCl2. In the initial few recordings, we confirm that the recorded current is glutamate by observing a near-complete blockade of evoked current upon 100 μM TBOA and 300 μM DHK application (Fig. S5h). The remaining current was completely abolished by superfusing 0.5 μM TTX, confirming it as a neuronal-evoked glutamate current (Fig. S5h). Each stimulation pulse was repeated 5 times (sweeps) and a minimum of two sweeps were averaged to compute the peak current and charge transfer after excluding the sweeps with baseline fluctuation or noise.

#### Depolarization evoked potassium uptake current:

To record depolarization evoked astrocytic potassium uptake current, we positioned the stimulator and patch pipette as described above and incubated slices in a mixture of 20μM Bicuculin, 50 μM D-AP-5, 20 μM CNQX, 100 μM TBOA, and 300 μM DKH. The stimulation protocol consisted of initial 0.1 mA and 0.2mA pulses followed by 0.5mA and 5 subsequent pulses with 0.5mA increment capping at 3mA (pulse duration 200μs). Each stimulation pulse was repeated 3 times (sweeps) and a minimum of two sweeps were averaged to compute the peak current and charge transfer after excluding the sweeps with baseline fluctuation or noise.

#### Astrocytic uptake of exogenously applied glutamate:

To measure the glutamate uptake capacity of astrocytes we adopted the exogenous glutamate puffing method as described previously with minor modifications[30, 34]. In brief, we constantly perfused slices with ACSF containing 500 nM TTX, 20 μM bicuculline, 100 μM CdCl2, 50 μM D-AP5, and 50 μM CNQX and 100 μM BaCl2. After patching an astrocyte, a 500 msec puff (2PSI pressure using a Pico-liter Injector PLI-10 from Warner Instruments) of 200 μM glutamate solution (120mM NaCl, 3.5mM KCl, 25mM HEPES, 10mM glucose, 200 μM Glutamate) was applied from a distance of ~100 μm by a 5–8mΩ open tip resistance glass pipette. In several random recordings, we applied a mixture of 100 μM TBOA and 300 μM DHK to confirm that the recorded current was glutamate (Fig. 6i). We recorded 5 sweeps and averaged a minimum of 3 sweeps to generate a result sweep that was utilized to compute the data. The sweeps with fluctuating baseline and noise were excluded from the analysis. The averaged trace of uptake current was analyzed using Clampfit 10.6 or Clampfit 11.2 program to generate the below-described measurements. The peak current was calculated by subtracting the baseline from the peak response. The total charge transfer was computed by calculating the total areas under the curve of glutamate uptake current response. Decay time and decay slope were calculated from the decaying phase (100 % to 37% of the peak) of the uptake current.

### Immunohistochemistry (IHC) and confocal imaging:

#### IHC:

Mice were injected with a mixture of ketamine and xylazine (100 mg/kg and 10 mg/kg, respectively) and subsequently perfused transcardially with PBS followed by 4% PFA. We dissected out the brains and stored them overnight in 4% PFA at 4 °C followed by storing them in PBS at 4 °C until sectioning was done. We cut 50-μm-thick floating sections using 5100MZ vibratome from Campden instruments or Pelco EasiSlicer from Ted Pella. The sections were either used for IHC immediately or stored at −20 °C in a custom-made storage medium (10% (v/v) 0.2mM phosphate buffer, 30% (v/v) glycerol, 30% (v/v) ethylenglycol in deionized water, pH 7.2–7.4) for future uses. To minimize procedure-associated variations, we stained duplicate sections from 5 to 7 mice of each experimental group in a single batch. In brief, sections were retrieved from −20 °C storage, rinsed thrice with PBS, and permeabilized and blocked by incubating them in blocking buffer (0.5% Triton X-100 and 10% goat serum in PBS) for 2 hr at RT in a 24-well plate. Sections were incubated for 18–24hrs at 4 °C with appropriate primary antibodies or biotinylated WFA (Cat# B-1355, Vector Laboratories) in diluted blocking buffer (1:3 of blocking buffer and PBS). Following this, we incubated sections with appropriate secondary antibodies and Alexa Fluor^®^ 555-conjugated streptavidin (Cat# S32355, ThermoFisher Scientific, 1:500) in diluted blocking buffer overnight at 4 °C in dark. Further, the sections were rinsed with PBS and were mounted on the glass slides (Fisherfinest 25 × 25 × 1, Cat# 12-544-2) covered with cover glass, and the edges of the slides were sealed with nail polish. The primary and secondary antibodies used are enlisted in Supplementary Table 1.

#### Confocal imaging:

Representative images and data in Figs. 1a–f, S1, 2a–e, S2, 3, S3, 4, S4, and S5 e–h, were acquired using Nikon A1 confocal microscope, and quantification was performed by associated NIS-Elements AR analysis program. Images and data in the remaining figures were acquired using Olympus FV 3000 confocal microscope and images were analyzed using the ImageJ program. We utilized several different objective lenses including 10x (air), 20x(air), 40x(oil), 60x (oil), or 100x (oil) with a range of optical zoom based on the experimental requirement. Images were acquired as 12 bits, and acquisition settings were minimally adjusted to accommodate a few unsaturated and saturated pixels.

### Quantification of IHC images:

#### PNN disruption analysis

The spread of PNN disruption by ChABC injection in mice brains was quantified from whole coronal section images (Fig. 4b). We drew uniform-sized ROIs (0.4×0.4 mm^2^) adjacent to each other starting from the ChABC incision site towards the lateral side of the coronal plane. The mean fluorescence intensity was computed. All analyzed images/ROIs at similar distances from the incision site were tabulated to plot the mean and SD of the fluorescence intensity. To assess the PNN disruption on PV cells after AAV-mediated Acan KO, we selected a 0^m perimeter area of cell soma and binarized the WFA signal using an automated thresholding method (OTSU) in ImageJ. WFA area from individual cells from different groups was utilized for plotting the graph (Fig. 5c).

#### PNN holes analysis:

We assessed the PNN holes for the presence of astrocytic processes (Fig. 1) and synaptic contacts (Fig. 2) and their fate after ChABC treatment (Fig. S4) using the PNN line intensity profile method with slight modifications as described previously[21, 22]. In brief, we acquired high magnification (200x or higher) images of individual PNNs at their maximum perimeter plane (Fig. 1a main image). Subsequently, we drew a polyline on PNN (WFA) along the entire periphery of the cell and generated an intensity profile consisting of high-intensity peaks (PNN-CSPGs) and low-intensity drops (PNN holes). We set a threshold of WFA intensity (ranging from 50–66% of the peak WFA intensity) that covered the maximum number of drops as PNN holes. The WFA intensity drops under the threshold (Fig. 1a, blue area in top graph) were considered holes. The presence of a specific fluorophore peak in the PNN hole was determined by the presence of a clearly distinguishable peak within the two consecutive peaks of WFA (Fig. 1a red areas in bottom graph). Subsequently, we provided unique identifying marks to each PNN hole and computed the presence or absence of astrocytic/synaptic components within it. To quantify the degree of perforations in PNNs after ChABC disruption, we counted the number of PNN holes as described above and normalized it to the cell perimeter. This method was utilized for analyzing PNN holes in Figs. 1, 2, S1, S2, and S4.

#### Quantification of PNN disruption and astrocytic Kir4.1 and Glt1 expressions in fixed ex-vivo acute brain slices:

To assess whether ChABC-mediated PNN digestion in acute brain slices alters the expressions of Kir4.1 and Glt1 to influence the astrocytic potassium and glutamate uptake, we fixed acute brain slices after electrophysiological recordings and performed immunostaining using specific antibodies and WFA. 40x magnification images were acquired using Olympus FV 3000 and analyzed using ImageJ. The signal of the individual channel (Kir4.1/Glt1, WFA, and AldheGFP) was binarized using an in-build thresholding function OTSU, and the resulting total area was tabulated to plot the graphs (Figs. 7h – k, S6).

#### Quantification of astrocytic coverage and synaptic puncta analysis:

To quantify the pericellular astrocytic coverage of PV / excitatory neurons, we acquired high-magnification images using either Nikon A1 (40×5 optical zoom oil immersion lens) or Olympus Fluoview FV 3000 (100×3 oil immersion objective lens) with a 0.2μm optical plane thickness (Fig. S3). After image acquisition, we generated a binary representation of the cell soma using inbuilt functions in ImageJ and Nikon elements programs. We defined a 0.8μm wide perimeter from the cell surface as a pericellular area (based on the measurement of the thickness of the PNN). Subsequently, we binarized the individual channels (AldheGFP / s100b / Glt1 / Kir4.1) using inbuilt auto thresholding functions in Nikon elements or ImageJ (OTSU). Using Boolean operations, we computed the binary areas of different astrocytic markers confined to the cell perimeter defined above (Fig. S3). We normalized the pericellular area with the perimeter of the same cell before pooling images from a section/mouse (Fig. 4). In Figs. 3d and 5 controls and experimental cells were from the same section, therefore we used individual cells data for plotting graphs and statistical tests.

We added one more step of *find maxima* in ImageJ or an analogous function in Nikon AR analysis programs to quantify the overall and pericellular numerical densities of vGlut1 and vGAT puncta in the above protocol (Fig. S3). A prominence setting of 500 (for vGlut1 puncta) or 2000 (for vGAT puncta) was found appropriate to capture all puncta and was used for images. The total number of synapses in the entire image was used to plot the total vGlut1/vGAT puncta (Fig. 4d – e). We used Boolean operations to compute the total pericellular synapses and pericellular synapses with astrocytic processes in contact with them. The resultant absolute values were normalized to the perimeter of the individual cells and were used for data pooling or directly for plotting graphs.

#### Volumetric analysis in Imaris:

The representative 3D reconstruction images of the PNN and pericellular astrocytic processes in Figs. 1, 2, and 4 were generated using Imaris v9.90 (Oxford Instruments). In brief, we generated volumetric masks from the PV channel that were expanded by 0.8μm – 1.0μm to accommodate pericellular PNN structures. These masks were then used to create new astrocytic and synaptic data channels that excluded structures outside of the pericellular domain. The enlarged PV channel volume was created using the surface creation tool with smoothing detail enabled and a surface grain size set to 0.103μm. Background subtraction was also enabled with the diameter of the largest sphere set to 0.388μm and manual thresholding set to a value of 200. Astrocytic and synaptic channel volumes were created using the surface creation tool with smoothing detail enabled and a surface grain size set to 0.103μm. Background subtraction was also enabled with the diameter of the largest sphere set to 0.388μm and manual thresholding set to a value of 200.

Illustrations / cartoons in Figs. 4, 5, 6, 7, and 8 were created with BioRender.com under a paid subscription.

### Statistical analysis

Data in the bar diagrams are expressed as mean ± SD unless stated otherwise in the specific figure. Individual data points are represented by dots. Figure legends contain the essential details including numerical values of mean, standard deviation, biological or technical replicates, statistical tests, and corrections. The detailed statistical analysis data including test statistics, P values, post-hoc comparisons, and corrections are summarized in Supplementary Table 2. The sample size was not predetermined. We ran appropriate normality tests and found that data distribution was sufficiently normal and variance within groups was sufficiently similar to be used for parametric tests. Therefore, experimental designs with two treatment groups were analyzed by two-tailed unpaired t-test unless stated otherwise in the figure legends. Welch’s correction was applied regardless of statistically different variances. Experimental designs with more than two groups were analyzed using one-way ANOVA or two-way ANOVA followed by Tukey’s post-hoc multiple comparison tests. Statistically significant difference between groups were notified in graphs using asterisk(s) (*P < 0.05, **P < 0.01, ***P < 0.001, ****P < 0.0001). Data analysis was performed using Microsoft Excel and Origin 2021 (OriginLab).

## Supplementary Material

1

## References

[1] Watanabe K., , Three-dimensional organization of the perivascular glial limiting membrane and its relationship with the vasculature: a scanning electron microscope study. Okajimas Folia Anat.Jpn., 2010. 87(3): p. 109–121. 21174940 10.2535/ofaj.87.109

[2] Araque A., , Tripartite synapses: glia, the unacknowledged partner. Trends in neurosciences, 1999. 22(5): p. 208–215. 10322493 10.1016/s0166-2236(98)01349-6

[3] Asztely F., Erdemli G., and Kullmann D.M., Extrasynaptic glutamate spillover in the hippocampus: dependence on temperature and the role of active glutamate uptake. Neuron, 1997. 18(2): p. 281–293. 9052798 10.1016/s0896-6273(00)80268-8

[4] Parsons M.P. and Raymond L.A., Extrasynaptic NMDA receptor involvement in central nervous system disorders. Neuron, 2014. 82(2): p. 279–293. 24742457 10.1016/j.neuron.2014.03.030

[5] Chaunsali L., Tewari B.P., and Sontheimer H., Perineuronal Net Dynamics in the Pathophysiology of Epilepsy. Epilepsy Currents, 2021. 21(4): p. 273–281. 34690566 10.1177/15357597211018688 PMC8512927

[6] Tewari B.P., , A glial perspective on the extracellular matrix and perineuronal net remodeling in the central nervous system. Frontiers in Cellular Neuroscience, 2022. 16: p. 1022754. 36339816 10.3389/fncel.2022.1022754 PMC9630365

[7] Frischknecht R., , Brain extracellular matrix affects AMPA receptor lateral mobility and short-term synaptic plasticity. Nature neuroscience, 2009. 12(7): p. 897–904. 19483686 10.1038/nn.2338

[8] Kochlamazashvili G., , The extracellular matrix molecule hyaluronic acid regulates hippocampal synaptic plasticity by modulating postsynaptic L-type Ca 2+ channels. Neuron, 2010. 67(1): p. 116–128. 20624596 10.1016/j.neuron.2010.05.030 PMC3378029

[9] Favuzzi E., , Activity-dependent gating of parvalbumin interneuron function by the perineuronal net protein brevican. Neuron, 2017. 95(3): p. 639–655. e10. 28712654 10.1016/j.neuron.2017.06.028

[10] Bal M., , Reelin mobilizes a VAMP7-dependent synaptic vesicle pool and selectively augments spontaneous neurotransmission. Neuron, 2013. 80(4): p. 934–46. 24210904 10.1016/j.neuron.2013.08.024 PMC3840105

[11] Wang X. b., , Extracellular proteolysis by matrix metalloproteinase-9 drives dendritic spine enlargement and long-term potentiation coordinately. Proceedings of the National Academy of Sciences, 2008. 105(49): p. 19520–19525. 10.1073/pnas.0807248105 PMC2614793 19047646

[12] Orlando C., , Perisynaptic chondroitin sulfate proteoglycans restrict structural plasticity in an integrin-dependent manner. Journal of Neuroscience, 2012. 32(50): p. 18009–18017. 23238717 10.1523/JNEUROSCI.2406-12.2012 PMC6621736

[13] Roszkowska M., , CD44: a novel synaptic cell adhesion molecule regulating structural and functional plasticity of dendritic spines. Mol Biol Cell, 2016. 27(25): p. 4055–4066. 27798233 10.1091/mbc.E16-06-0423 PMC5156546

[14] de Vivo L., , Extracellular matrix inhibits structural and functional plasticity of dendritic spines in the adult visual cortex. Nature Communications, 2013. 4(1): p. 1484. 10.1038/ncomms2491 23403561

[15] Sonntag M., , Synaptic coupling of inner ear sensory cells is controlled by brevican-based extracellular matrix baskets resembling perineuronal nets. BMC Biology, 2018. 16(1): p. 99. 30253762 10.1186/s12915-018-0566-8 PMC6156866

[16] Brückner G., , Extracellular matrix organization in various regions of rat brain grey matter. Journal of neurocytology, 1996. 25(1): p. 333–346. 8818977 10.1007/BF02284806

[17] Fawcett J.W., Oohashi T., and Pizzorusso T., The roles of perineuronal nets and the perinodal extracellular matrix in neuronal function. Nature Reviews Neuroscience, 2019. 20(8): p. 451–465. 31263252 10.1038/s41583-019-0196-3

[18] Pizzorusso T., , Reactivation of ocular dominance plasticity in the adult visual cortex. Science, 2002. 298(5596): p. 1248–1251. 12424383 10.1126/science.1072699

[19] Torres-Ceja B. and Olsen M.L., A closer look at astrocyte morphology: Development, heterogeneity, and plasticity at astrocyte leaflets. Curr Opin Neurobiol, 2022. 74: p. 102550. 35544965 10.1016/j.conb.2022.102550 PMC9376008

[20] Cahoy J.D., , A transcriptome database for astrocytes, neurons, and oligodendrocytes: a new resource for understanding brain development and function. J Neurosci, 2008. 28(1): p. 264–78. 18171944 10.1523/JNEUROSCI.4178-07.2008 PMC6671143

[21] Tewari B.P. and Sontheimer H., Protocol to Quantitatively Assess the Structural Integrity of Perineuronal Nets ex vivo. Bio-protocol, 2019. 9(10): p. e3234–e3234. 33654764 10.21769/BioProtoc.3234 PMC7854210

[22] Tewari B.P., , Perineuronal nets decrease membrane capacitance of peritumoral fast spiking interneurons in a model of epilepsy. Nature Communications, 2018. 9(1): p. 4724. 10.1038/s41467-018-07113-0 PMC6226462 30413686

[23] Carceller H., , Perineuronal Nets: Subtle Structures with Large Implications. Neuroscientist, 2022: p. 10738584221106346. 10.1177/10738584221106346 35872660

[24] Yang Y., Higashimori H., and Morel L., Developmental maturation of astrocytes and pathogenesis of neurodevelopmental disorders. Journal of Neurodevelopmental Disorders, 2013. 5(1): p. 22. 23988237 10.1186/1866-1955-5-22 PMC3765765

[25] Bruckner G., , Postnatal development of perineuronal nets in wild-type mice and in a mutant deficient in tenascin-R. J Comp Neurol, 2000. 428(4): p. 616–29. 11077416 10.1002/1096-9861(20001225)428:4<616::aid-cne3>3.0.co;2-k

[26] Carstens K.E., , Perineuronal nets suppress plasticity of excitatory synapses on CA2 pyramidal neurons. Journal of Neuroscience, 2016. 36(23): p. 6312–6320. 27277807 10.1523/JNEUROSCI.0245-16.2016 PMC4899529

[27] Rowlands D., , Aggrecan directs extracellular matrix-mediated neuronal plasticity. Journal of Neuroscience, 2018. 38(47): p. 10102–10113. 30282728 10.1523/JNEUROSCI.1122-18.2018 PMC6596198

[28] Carceller H., , Perineuronal Nets Regulate the Inhibitory Perisomatic Input onto Parvalbumin Interneurons and γ Activity in the Prefrontal Cortex. Journal of Neuroscience, 2020. 40(26): p. 5008–5018. 32457072 10.1523/JNEUROSCI.0291-20.2020 PMC7314408

[29] Christensen A.C., , Perineuronal nets stabilize the grid cell network. Nature Communications, 2021. 12(1): p. 253. 10.1038/s41467-020-20241-w PMC7801665 33431847

[30] Somaiya R.D., , Development of astrocyte morphology and function in mouse visual thalamus. Journal of Comparative Neurology, 2022. 530(7): p. 945–962. 34636034 10.1002/cne.25261 PMC8957486

[31] Campbell S.L., Hablitz J.J., and Olsen M.L., Functional changes in glutamate transporters and astrocyte biophysical properties in a rodent model of focal cortical dysplasia. Frontiers in Cellular Neuroscience, 2014. 8(425). 10.3389/fncel.2014.00425 PMC4269128 25565960

[32] Bergles D.E. and Jahr C.E., Synaptic Activation of Glutamate Transporters in Hippocampal Astrocytes. Neuron, 1997. 19(6): p. 1297–1308. 9427252 10.1016/s0896-6273(00)80420-1

[33] Campbell S.C., , Potassium and glutamate transport is impaired in scar-forming tumor-associated astrocytes. Neurochemistry International, 2020. 133: p. 104628. 31825815 10.1016/j.neuint.2019.104628 PMC6957761

[34] Robel S., , Reactive astrogliosis causes the development of spontaneous seizures. Journal of Neuroscience, 2015. 35(8): p. 3330–3345. 25716834 10.1523/JNEUROSCI.1574-14.2015 PMC4339349

[35] Sorg B.A., , Casting a Wide Net: Role of Perineuronal Nets in Neural Plasticity. J Neurosci, 2016. 36(45): p. 11459–11468. 27911749 10.1523/JNEUROSCI.2351-16.2016 PMC5125213

[36] Yong V.W., , Metalloproteinases in biology and pathology of the nervous system. Nature Reviews Neuroscience, 2001. 2(7): p. 502. 11433375 10.1038/35081571 PMC7097548

[37] Gottschall P.E. and Howell M.D., ADAMTS expression and function in central nervous system injury and disorders. Matrix Biology, 2015. 44: p. 70–76. 25622912 10.1016/j.matbio.2015.01.014 PMC5068130

[38] Morawski M., , Ion exchanger in the brain: quantitative analysis of perineuronally fixed anionic binding sites suggests diffusion barriers with ion sorting properties. Scientific reports, 2015. 5. 10.1038/srep16471 PMC4664884 26621052

[39] Dubey D., , Increased metalloproteinase activity in the hippocampus following status epilepticus. Epilepsy Research, 2017. 132: p. 50–58. 28292736 10.1016/j.eplepsyres.2017.02.021 PMC6690398

[40] McRae P.A. and Porter B.E., The perineuronal net component of the extracellular matrix in plasticity and epilepsy. Neurochemistry international, 2012. 61(7): p. 963–972. 22954428 10.1016/j.neuint.2012.08.007 PMC3930202

[41] Gonzalez-Burgos G., Fish K.N., and Lewis D.A., GABA Neuron Alterations, Cortical Circuit Dysfunction and Cognitive Deficits in Schizophrenia. Neural Plasticity, 2011. 2011: p. 723184. 21904685 10.1155/2011/723184 PMC3167184

[42] Brückner G., Morawski M., and Arendt T., Aggrecan-based extracellular matrix is an integral part of the human basal ganglia circuit. Neuroscience, 2008. 151(2): p. 489–504. 18055126 10.1016/j.neuroscience.2007.10.033

[43] Gittis Aryn H., , Rapid Target-Specific Remodeling of Fast-Spiking Inhibitory Circuits after Loss of Dopamine. Neuron, 2011. 71(5): p. 858–868. 21903079 10.1016/j.neuron.2011.06.035 PMC3170520

[44] Dityatev A., Seidenbecher C.I., and Schachner M., Compartmentalization from the outside: the extracellular matrix and functional microdomains in the brain. Trends Neurosci, 2010. 33(11): p. 503–12. 20832873 10.1016/j.tins.2010.08.003

[45] Dityatev A. and Rusakov D.A., Molecular signals of plasticity at the tetrapartite synapse. Current Opinion in Neurobiology, 2011. 21(2): p. 353–359. 21277196 10.1016/j.conb.2010.12.006 PMC3368316

[46] Freeman M.R., Specification and morphogenesis of astrocytes. Science, 2010. 330(6005): p. 774–778. 21051628 10.1126/science.1190928 PMC5201129

[47] Hanson E., Danbolt N.C., and Dulla C.G., Astrocyte membrane properties are altered in a rat model of developmental cortical malformation but single-cell astrocytic glutamate uptake is robust. Neurobiol Dis, 2016. 89: p. 157–68. 26875663 10.1016/j.nbd.2016.02.012 PMC4794447

