# Supplementary material for: Perineuronal nets support astrocytic ion and glutamate homeostasis at tripartite synapses
Source: Res Sq. 2023 Feb 2:rs.3.rs-2501039. Preprint. [Version 1] doi: 10.21203/rs.3.rs-2501039/v1 (PMC9915772; doi:10.21203/rs.3.rs-2501039/v1)
Supplement: 1 [file NIHPPRS2501039V1-supplement-1.pdf]

Supplementary figures

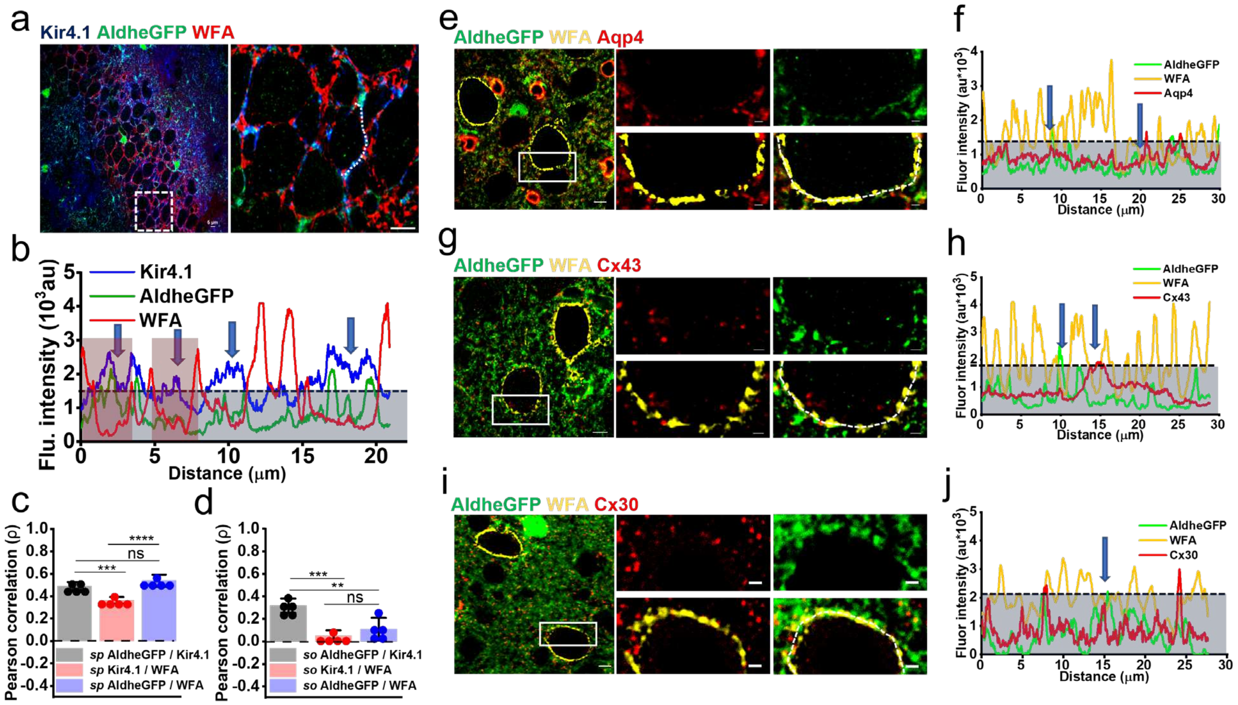

Fig. S1 In CA2 of hippocampus, PNNs holes contain astrocytic processes.

a Representative confocal micrographs showing hippocampal CA2 PNNs (WFA - red) and astrocytes labelled with AldheGFP (green) and Kir4.1 (blue). The right image shows the magnified area of the white square in left large image. Scale bar 5µm.

b Line intensity profiles of the white dotted line drawn in Fig. **a** (right), showing fluorescence intensity of PNN and astrocytic markers. Blue arrows point to the PNN holes occupied by astrocytic processes expressing Kir4.1. Red bars in between two consecutive WFA peaks represent the area of PNN holes wherein astrocytic processes can be confined.

c-d Bar diagrams showing Pearson correlation of spatial overlap between astrocytic markers AldheGFP and Kir4.1 with each other and with PNN marker WFA in **c** stratum pyramidale (Kir4.1-AldheGFP 0.49 ± 0.03, Kir4.1-WFA 0.36 ± 0.02, AldheGFP-WFA 0.53 ± 0.05) and **d** stratum radiatum of CA2 (Kir4.1-AldheGFP 0.31 ± 0.06, Kir4.1-WFA 0.05 ± 0.04, AldheGFP-WFA 0.10 ± 0.10). n = 5sections/5mice, ****P < 0.0001, ***P < 0.001, **P < 0.01, *P < 0.05, ns = P > 0.05. One-way ANOVA, Tukey's post-hoc test. Bar data are expressed as mean±SD; dots on the bars represent the individual data points.

e Representative confocal micrographs showing expression of aquaporin 4 (red) in astrocytic processes (AldheGFP – green) in holes of cortical PNNs (WFA - yellow). The right side panels show the magnified area marked by a white rectangle in the left image.

f Intensity profiles of the white dotted line drawn on the PNN (bottom right panel) represented PNN holes (marked by blue arrows) occupied with astrocytic processes (AldheGFP-green) expressing Aqp4 (red).

g Representative confocal micrographs showing expression of connexin 43 (red) in astrocytic processes (AldheGFP – green) in holes of cortical PNNs (WFA - yellow). The right side panels show the magnified area marked by the white rectangle in the left image.

h Intensity profiles of the white dotted line drawn on the PNN (bottom right panel) represented PNN holes (marked by blue arrows) occupied with astrocytic processes (AldheGFP-green) expressing connexin 43 (red).

i Representative confocal micrographs showing expression of connexin 30 (red) in astrocytic processes (AldheGFP – green) in holes of cortical PNNs (WFA - yellow). The right side panels show a magnified area marked by the white rectangle in left image.

j Intensity profiles of the white dotted line drawn on the PNN (bottom right panel) represented PNN holes (marked by blue arrows) occupied with astrocytic processes (AldheGFP-green) expressing connexin 30 (red).

Scale bars 5μm in large images, 1μm in magnified images in e-i. Blue area under the dotted lines in line profiles in b, f, h, and j represents the WFA threshold.

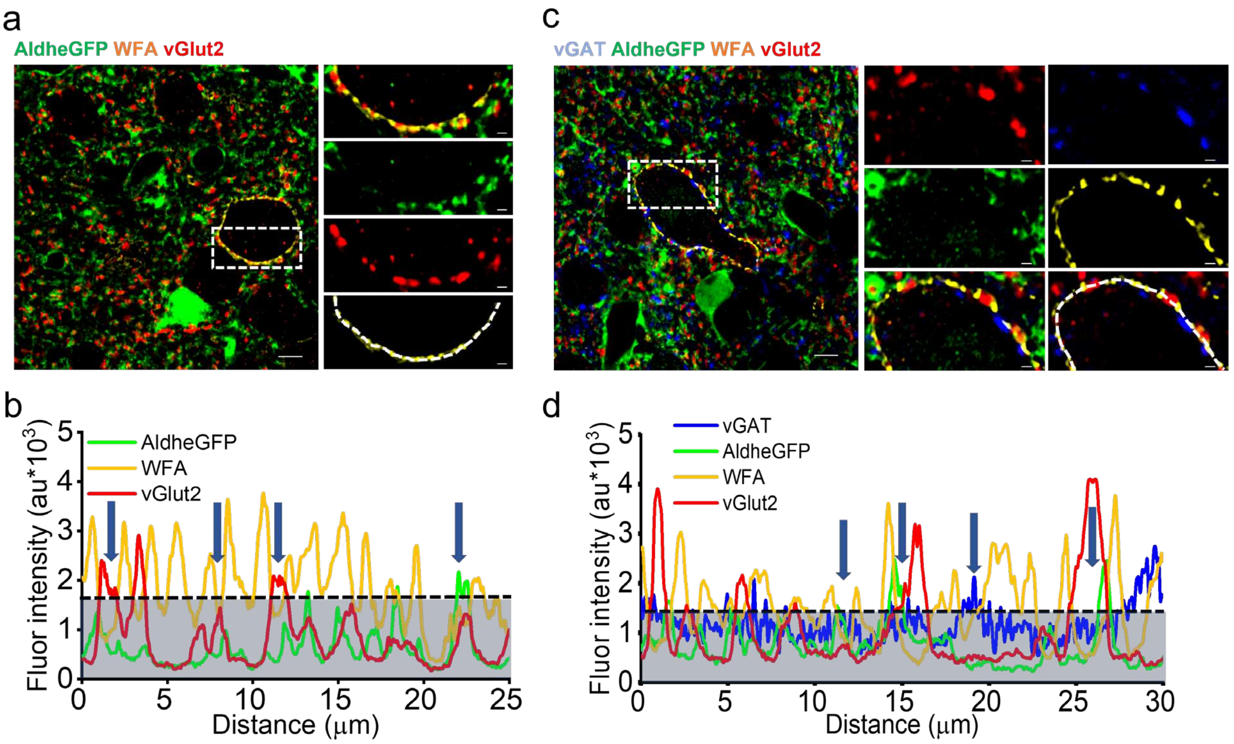

Fig. S2 PNN holes contain astrocytic processes and thalamocortical synaptic contacts.

a Representative confocal micrographs showing expression of vGlut2 (red) expressing synapses as well as astrocytic processes (AldheGFP – green) in holes of cortical PNNs (WFA - yellow). Right side panels show a magnified area marked by the white rectangle in left image.

b Intensity profiles of the white dotted line drawn on the PNN (bottom right panel) represented PNN holes (marked by blue arrows) occupied with astrocytic processes (AldheGFP-green) and vGlut2 expressing synapses (red).

c Representative confocal micrographs showing expression of glutamatergic (vGlut2 - red) and GABAergic (vGAT - blue) synapses as well as astrocytic processes (AldheGFP – green) in holes of cortical PNNs (WFA - yellow). Right side panels show a magnified area marked by the white rectangle in left image.

d Intensity profiles of the white dotted line drawn on the PNN (bottom right panel) represented PNN holes (marked by blue arrows) occupied with astrocytic processes (AldheGFP-green) and glutamatergic (vGlut2 - red) and GABAergic (vGAT - blue) synapses.

Scale bar 5μm in large images, 1μm in magnified images in both a and c. Blue area under the dotted lines in line profiles in b and d represents the WFA threshold.

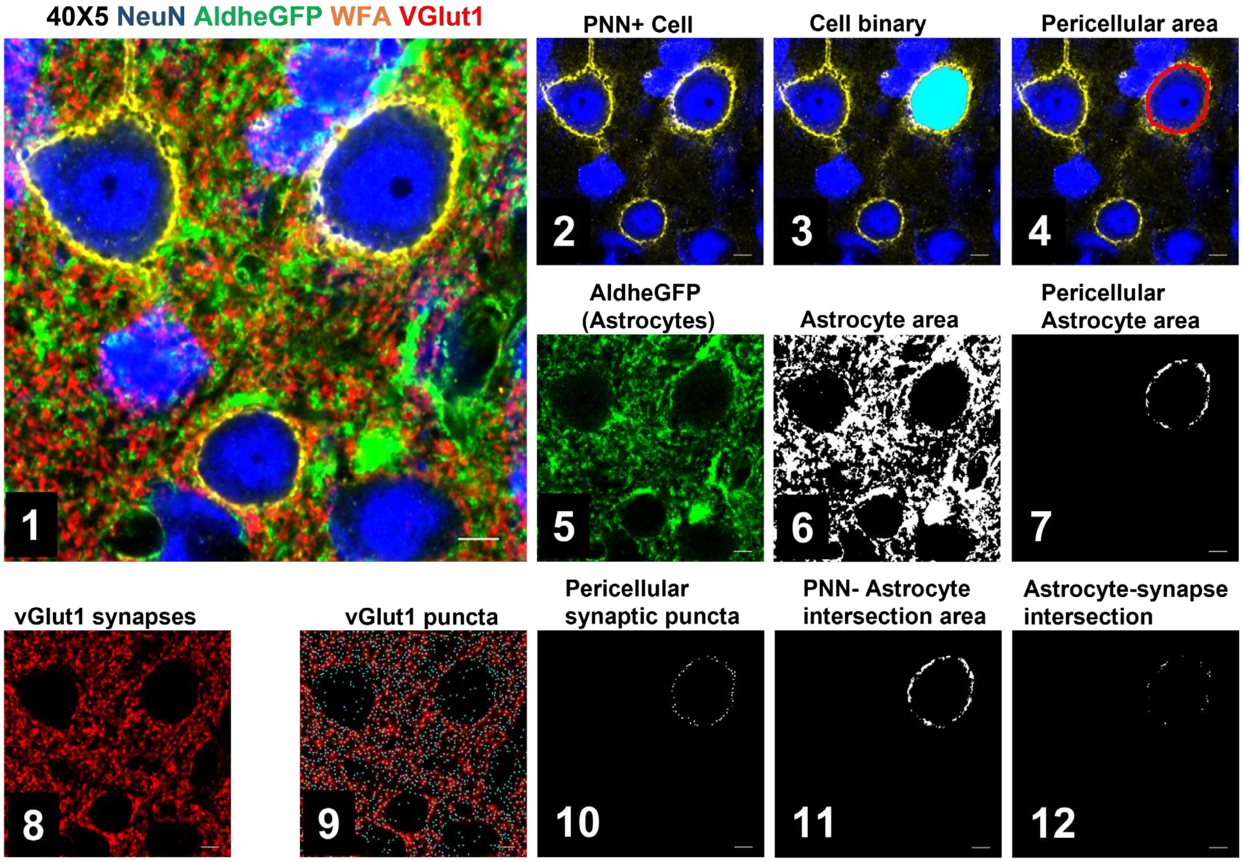

Fig. S3 Pericellular astrocytic coverage and synaptic contacts analysis method.

Multichannel confocal image (1), showing immunofluorescence labeling of neurons (NeuN), astrocytes (AldheGFP), PNNs (WFA), and synaptic terminals (vGlut1). The NeuN signal of the soma of PNN-expressing neurons (2), is binarized (3), and the pericellular 0.8 - 1µm area (4) is defined. AldheGFP signal (5), is binarized using automated OTSU function (6), and pericellular AldheGFP area (7), is extracted by intersecting (4) and (6) binary images. Synaptic marker vGlut1 (8), image is processed with an automated peak detection function to detect vGlut1 puncta (8). Intersecting (9) with (4) generates pericellular synaptic puncta (10). Intersecting the PNN signal with (7) generates a PNN-astrocyte intersection area (11), and intersecting (7) with (10) generates vGlut1 puncta in contact with the pericellular astrocytic area (12). Scale bar: 5µm.

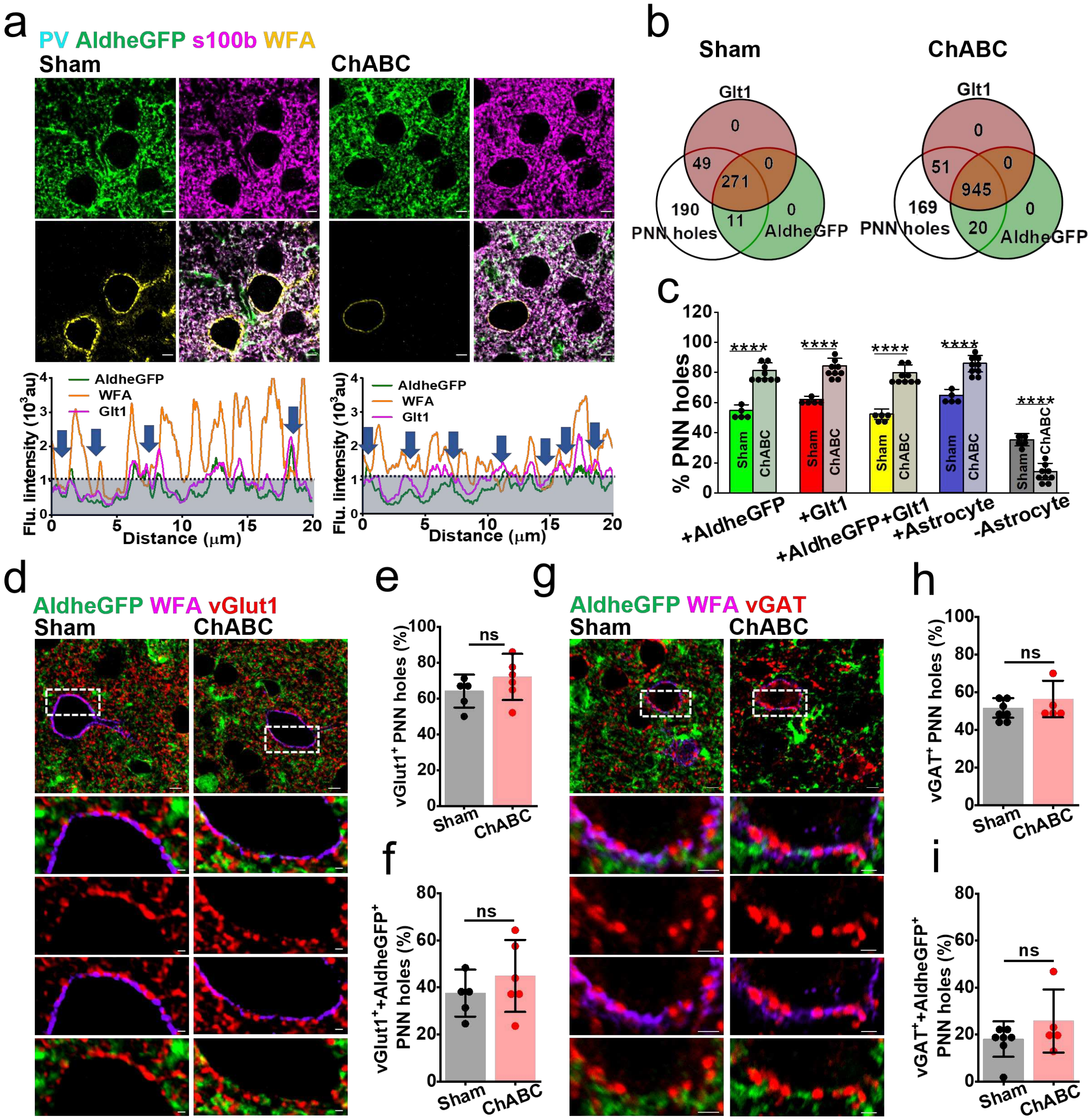

Fig. S4 Astrocytic processes occupy newly formed PNN holes after ChABC treatment.

a Confocal micrographs showing immunofluorescence of astrocytes (AldheGFP – green), astrocytic glutamate transporter Glt1 (magenta), and PNNs (WFA - yellow), from sham and ChABC-injected mouse cerebral cortex. Scale bar 5μm. Line intensity profiles of a typical PNN from sham (left) and ChABC treated (right) conditions showing low WFA intensity and high occupancy of PNN perforations with astrocytic processes. Blue area under the dotted line represents the threshold WFA intensity.

b Venn diagrams showing the proportional occupancy of PNN holes by astrocytic processes (AldheGFP + Glt1) in sham (left) and ChABC-treated (right) conditions.

c Bar diagram showing the percent of total PNN holes in sham and ChABC treated groups occupied by AldheGFP (Control 54.76 ± 3.75, ChABC 81.05 ± 5.24), Glt1 (Control 62.09 ± 2.16, ChABC 84.15 ± 5.26) and both (Control 52.32 ± 3.50, ChABC 79.64 ± 5.38), any astrocytic marker positive (Control 64.67 ± 4.09, ChABC 85.80 ± 5.51) and any astrocytic marker negative (Control 35.32 ± 4.09, ChABC 14.19 ± 5.51) holes. n ≥40 PNNs/8s/4m in each group.

d Confocal micrographs showing immunofluorescence of astrocytes (AldheGFP – green), excitatory terminals vGlut1 (red), and PNNs (WFA - magenta), from sham and ChABC-injected mouse brains. Magnified images of different combinations showing synaptic contacts in PNN holes in sham and ChABC-injected mouse brains. Scale bar 5µm in the large image, 1µm in magnified images.

e Pericellular density of vGlut1 terminal in the PNN holes in the ChABC-treated group remained unaltered compared to sham (Control 64.10 ± 9.14, n = 22PNNs/5m, ChABC 71.98 ± 12.89, 35PNNs/5m).

f Pericellular density of vGlut1 terminal with astrocytic contacts in the PNN holes in ChABC treated group remained unaltered compared to sham (Control 37.58 ± 9.97, n = 22PNNs/5m ChABC 44.87 ± 15.29, 35PNNs/5m).

g Confocal micrographs showing immunofluorescence of astrocytes (AldheGFP – green), inhibitory terminals vGAT (red), and PNNs (WFA – magenta), from sham and ChABC-injected mouse brains. Magnified images of different combinations showing synaptic contacts in PNN holes in sham and ChABC-injected mouse brains. Scale bar 5µm in the large image, 1µm in magnified images.

h Pericellular density of vGAT terminal in the PNN holes in ChABC treated group remained unaltered compared to sham (Control 51.62 ± 5.20, n = 40PNNs/6m; ChABC 56.36 ± 9.69, 26PNNs/5m).

i Pericellular density of vGAT terminal with astrocytic contacts in the PNN holes in ChABC treated group remained unaltered compared to sham (Control 18.11±7.53, n = 22PNNs/5m ChABC 25.81 ± 13.43, 35PNNs/5m).

s and m indicate the number of sections and mice respectively. ****P < 0.0001, ***P < 0.001, **P < 0.01, *P < 0.05, ns = P > 0.05. One-way ANOVA, Tukey's post-hoc test in d; Unpaired two-tailed t-test with Welch's correction in f, g, i, and j. Bar data are expressed as mean±SD; dots represent individual data points.

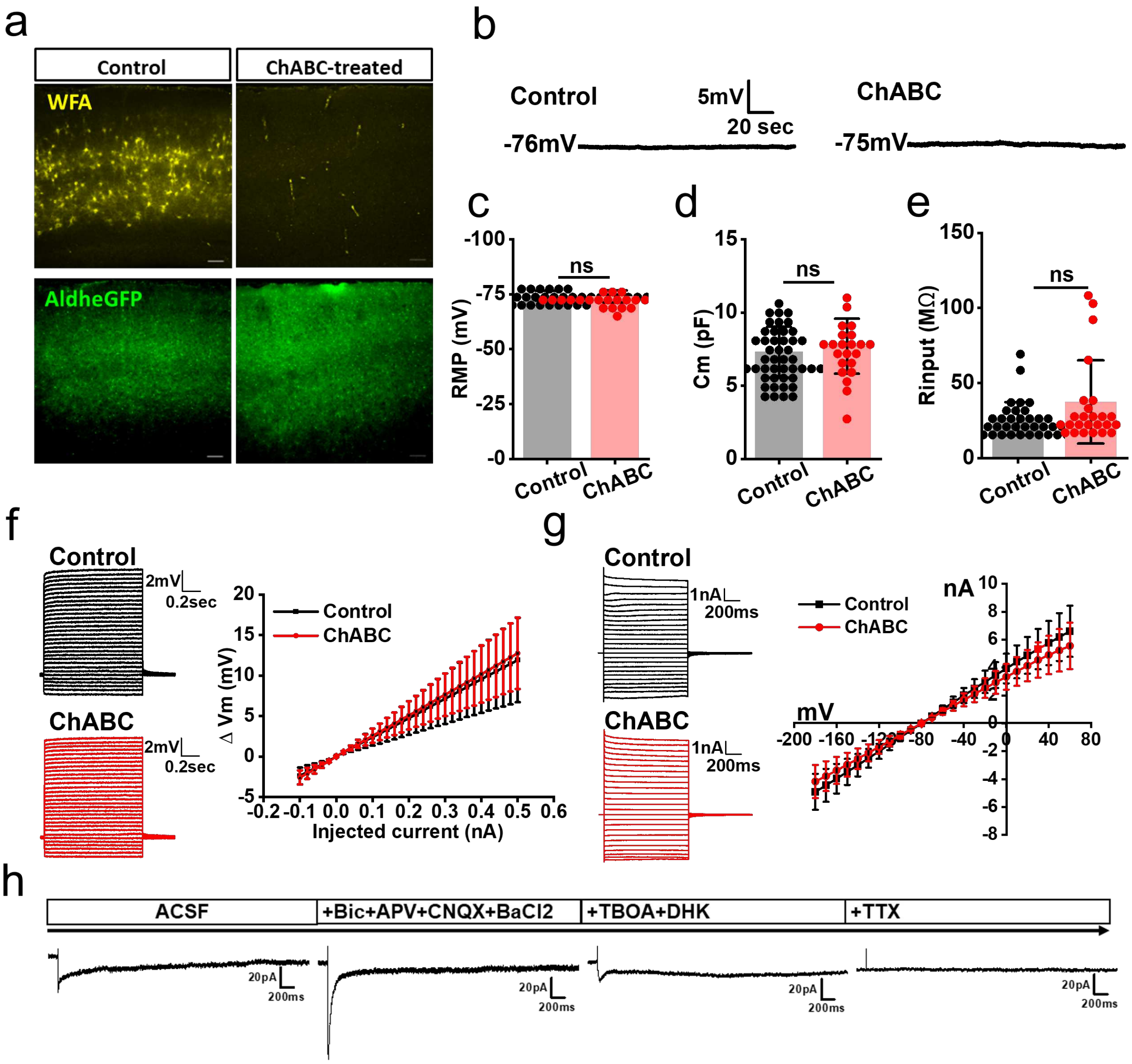

Figure S5. Biophysical properties of astrocytes remain unchanged on PNN disruption with ChABC.

a Confocal images of WFA and AldheGFP fluorescence in control and ChABC-treated acute slices fixed and stained after electrophysiological recordings. Scale 100µm.

b Representative current-clamp traces of astrocytic resting membrane potential from control and ChABC-treated slices.

c - e Bar diagrams showing unchanged (**c**) resting membrane potential (control -74.47 ± 2.24mV, n = 49c/18m; ChABC -74.04 ± 2.7mV, 24c/6m), (**d**) membrane capacitance (control 7.29 ± 1.3pF, n = 46c/17m, ChABC 7.70 ± 1.8, n = 22c/7m), and (**e**) input resistance (control -26.29 ± 37.46mΩ, n = 41c/18m; ChABC -37.46 ± 27.69 mΩ, 25c/7m) of astrocytes in ChABC treated slices compared to control slices. ns signifies P >0.05, Unpaired two-tailed student's t-test with Welch correction in **c** - **e**.

f Representative current clamp traces and IV plot showing the current-voltage relationship of astrocytes in control (n = 49c/18m) and ChABC treated (n = 28c/9m) slices.

g Representative voltage clamp traces, and IV plot showing the current-voltage relationship of astrocytes in control (n = 59c/18m) and ChABC treated (n = 38c/8m) slices.

h Representative voltage clamp traces of synaptically evoked currents in astrocytes in presence of different blockers to isolate glutamate current.

c and m represent the number of cells and mice respectively.

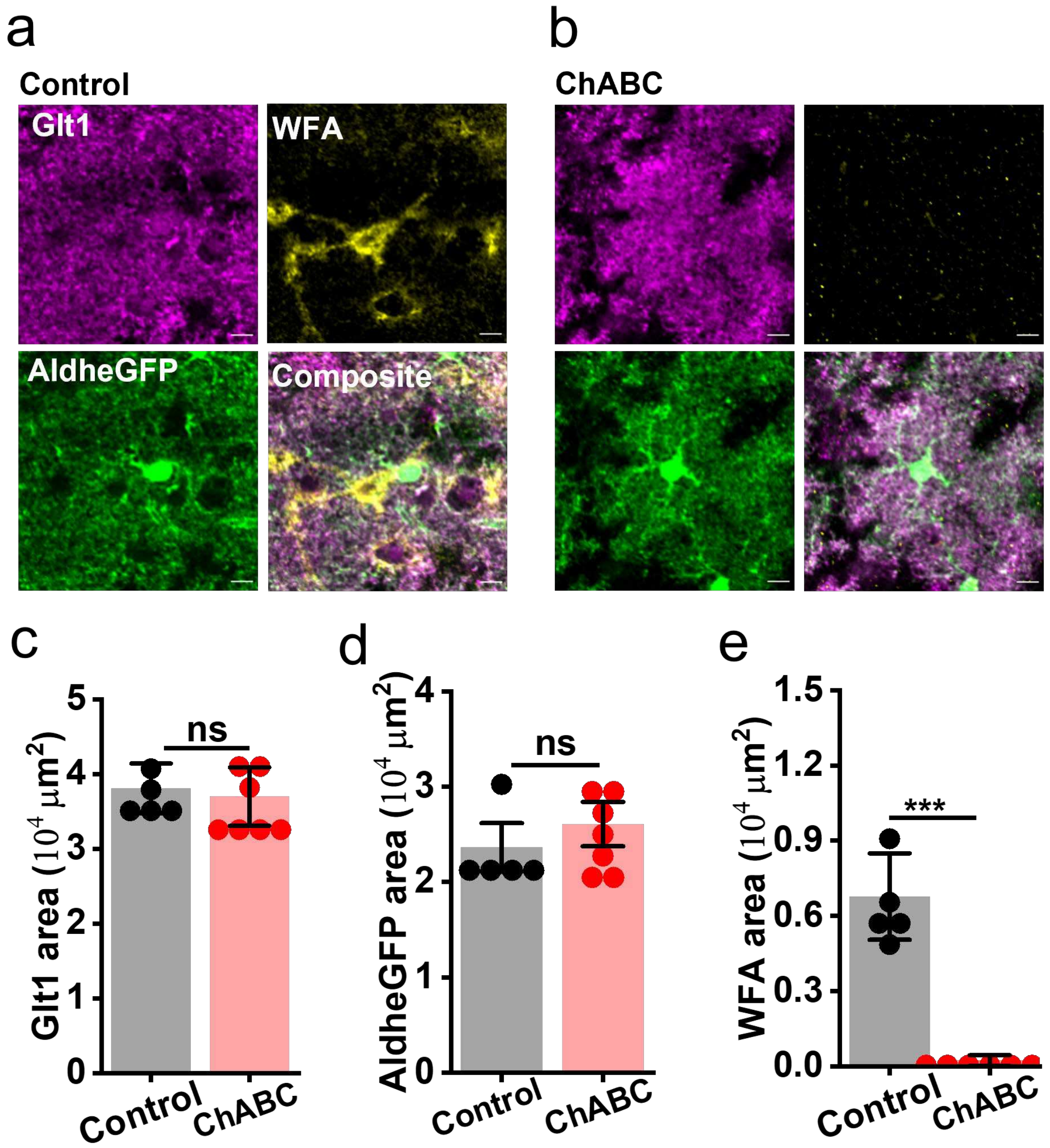

Figure S6. Unaltered glutamate transporter expression on PNN depletion in acute brain slices.

a - b Confocal micrographs of Glt1 (magenta), aldheGFP (green), and WFA (yellow) fluorescence from fixed acute slices from control (**a**) and after ChABC treatment (**b**). Scale bar 10µm.

c-e Bar diagrams of immunofluorescence area of, (**c**) Glt1 (control 38077.65 ± 3342.90; ChABC 36986.21 ± 3914.77), (**d**) AldheGFP (control 23635.67 ± 3772.71, ChABC 26070.47 ± 4100.87), and (**e**) WFA (control 6769.05 ± 1723.79, ChABC 226.70 ± 214.73) showing PNN disruption without any changes in astrocytic Glt1 expression. Control n = 5s/3m; ChABC 7s/3m in c-e. Units, µm^2 in c-e.

s and m represent the number of slices and mice respectively. ****$P < 0.0001$,***$P < 0.001$, **$P < 0.01$, *$P < 0.05$, ns = $P > 0.05$. unpaired two-tailed student t-test (equal variance not assumed). Bar data are expressed as mean±SD; dots on the bars represent the individual data points.
